# Exosomes in the nose induce immune cell trafficking and harbour an altered protein cargo in chronic airway inflammation

**DOI:** 10.1186/s12967-016-0927-4

**Published:** 2016-06-20

**Authors:** Cecilia Lässer, Serena E. O’Neil, Ganesh V. Shelke, Carina Sihlbom, Sara F. Hansson, Yong Song Gho, Bo Lundbäck, Jan Lötvall

**Affiliations:** Krefting Research Centre, Department of Internal Medicine and Clinical Nutrition, Sahlgrenska Academy, University of Gothenburg, Gothenburg, Sweden; Proteomics Core Facility, Sahlgrenska Academy, University of Gothenburg, Gothenburg, Sweden; Department of Life Sciences, Pohang University of Science and Technology, Pohang, Republic of Korea

**Keywords:** Asthma, Chronic rhinosinusitis, Exclusion list, Exosomes, Extracellular vesicles, Cell migration, Mass spectrometry, Nasal lavage fluid, Proteomics, Tandem mass tags

## Abstract

**Background:**

Exosomes are nano-sized extracellular vesicles participating in cell-to-cell communication both in health and disease. However, the knowledge about the functions and molecular composition of exosomes in the upper airways is limited. The aim of the current study was therefore to determine whether nasal exosomes can influence inflammatory cells and to establish the proteome of nasal lavage fluid-derived exosomes in healthy subjects, as well as its alterations in individuals with chronic airway inflammatory diseases [asthma and chronic rhinosinusitis (CRS)].

**Methods:**

Nasal lavage fluid was collected from 14 healthy subjects, 15 subjects with asthma and 13 subjects with asthma/CRS. Exosomes were isolated with differential centrifugation and the proteome was analysed by LC–MS/MS with the application of two exclusion lists as well as using quantitative proteomics. Ingenuity Pathways Analysis and GO Term finder was used to predict the functions associated with the exosomal proteome and a migration assay was used to analyse the effect on immune cells by nasal exosomes.

**Results:**

Firstly, we demonstrate that nasal exosomes can induce migration of several immune cells, such as monocytes, neutrophils and NK cells in vitro. Secondly, a mass spectrometry approach, with the application of exclusion lists, was utilised to generate a comprehensive protein inventory of the exosomes from healthy subjects. The use of exclusion lists resulted in the identification of ~15 % additional proteins, and increased the confidence in ~20 % of identified proteins. In total, 604 proteins were identified in nasal exosomes and the nasal exosomal proteome showed strong associations with immune-related functions, such as immune cell trafficking. Thirdly, a quantitative proteomics approach was used to determine alterations in the exosome proteome as a result of airway inflammatory disease. Serum-associated proteins and mucins were more abundant in the exosomes from subjects with respiratory diseases compared to healthy controls while proteins with antimicrobial functions and barrier-related proteins had decreased expression.

**Conclusions:**

Nasal exosomes were shown to induce the migration of innate immune cells, which may be important as the airway epithelium is the first line of defence against pathogens and allergens. The decreased expression in barrier and antimicrobial exosomal proteins in subjects with airway diseases, could possibly contribute to an increased susceptibility to infections, which have important clinical implications in disease progression.

**Electronic supplementary material:**

The online version of this article (doi:10.1186/s12967-016-0927-4) contains supplementary material, which is available to authorized users.

## Background

Nano-sized extracellular vesicles, called exosomes, are released by all cells, and contain multiple functional molecules that can be transferred from one cell to another, thereby influencing the recipient cell phenotype. The presence of exosomes within the lungs has been previously documented [1], and in 2011 we showed the presence of exosomes in the nasal cavity [2]. Bronchoalveolar lavage fluid (BALF) derived exosomes from asthmatic subjects has been shown to influence leukotriene production in bronchial epithelial cells [3], as well as displaying an altered miRNA profile compared to healthy subjects [4], however there is no published information about the functionality of exosomes present in the nasal cavity.

Exosomes in the nasal cavity are important to study in relation to chronic inflammatory processes, as the exceptional filtering capacity of the nose makes it a first line of defence against inhaled particles, such as dust, allergens and air pollution. Diseases of the airways can alter the epithelium and lead to impaired barrier defence function [5]. Respiratory diseases such as rhinitis, asthma and chronic rhinosinusitis (CRS) are common with prevalence’s of 27.5, 8.5 and 10.9 % respectively [6–8]. These diseases are intricately connected, with the severity of asthma being related to the degree of nasal symptoms [9]. However, it is not known whether exosomes in the nose are altered during chronic airway inflammatory diseases such as CRS and asthma, which is of particular interest as this is the site of inflammation.

We hypothesize that exosomes in the nasal cavity have biological functions, and that their molecular components are changed among individuals with different respiratory diseases. The aim of the current study was therefore to determine whether nasal exosomes can induce an inflammatory cell migratory phenotype, and to relate any such function with the nasal exosomal proteome. Additionally, we aimed to determine whether the nasal exosomal proteome is altered in patients with signs of chronic rhinosinusitis and asthma. The baseline nasal exosomal proteome in healthy individuals was determined utilising a dynamic exclusion LC–MS/MS approach, while the nasal exosomal proteome in subjects with asthma and CRS was compared to controls using a quantitative proteomics approach with tandem mass tags (TMT^®^).

## Methods

### Study subjects

**Fig. 1:**
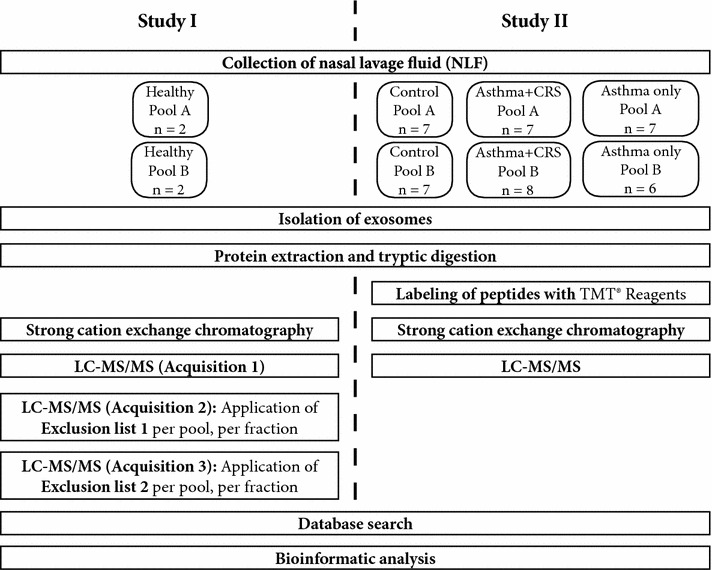
Schematic overview of the experimental workflow for the mass spectrometry part of study I and II. In study I, the protein content of exosomes from healthy individuals was analysed. Protein extracted from the isolated exosomes was subjected to strong cation exchange chromatography fractionation before analysis with a nano LC–MS/MS instrument. After the first acquisition all identified peptides were used to construct an exclusion list that was applied during the second acquisition. This was repeated for the third acquisition. Study II aimed to explore quantitative differences in the protein content of exosomes from respiratory diseases. The digested peptides were labelled with TMT reagents and subjected to fractionation before being analysed by nano LC–MS/MS. The resulting spectra from both study I and study II were searched in a database for identities and the reporter ions from the TMT reagents were used to quantify proteins in study II. All datasets were analysed with Ingenuity Pathways Analysis and GO Term Finder to identify enriched and associated cellular components, biological functions and processes

Two separate studies were conducted, study I and II. Study I was performed to determine the baseline protein cargo of nasal exosomes in healthy subjects and study II was performed to determine the alteration in the exosomal proteome during chronic airway inflammation by quantitative proteomics. Study I participants were five healthy non-smoking female subjects, with no current asthma or nasal symptoms. Four subjects were used to create two pools, pool A and pool B, with two subjects per pool (Fig. 1, study I). Samples from the fifth subject were used for validation experiments. In addition to the samples used for the baseline proteome, additional samples were obtained from the four subjects in the two pools for validation experiments.

Study II participants were selected from questionnaire respondents in the West Sweden Asthma Study [6], that had undergone a clinical examination at the Krefting Research Centre in Gothenburg. The clinical examination included skin prick tests, fraction of exhaled nitric oxide (FeNO), spirometry and a methacholine challenge. Study participants attending the clinical examination and fulfilling inclusion criteria described below, were invited to participate in a study on asthma and chronic rhinosinusitis (CRS) where several clinical samples were collected, including nasal lavage fluid (NLF). Several groups of subjects were analysed in this study; healthy controls (*controls*) (n = 9), subjects with asthma (*asthma only*) (n = 13), and subjects with both asthma and CRS (*asthma* + *CRS*) (n = 15). As the control group of nine subjects was too small to divide into two pools, the five healthy subjects in study I underwent the clinical examination and was also included as controls in study II, giving 14 subjects in the control group. Two pools per group was created; control pool A (n = 7), control pool B (n = 7), asthma only pool A (n = 7), asthma only pool B (n = 6), asthma + CRS Pool A (n = 7) and asthma + CRS Pool B (n = 8) (Fig. 1, study II). Subjects with CRS were defined as those meeting the nasal symptoms outlined in the EPOS2012 definition of CRS [10]. That is, subjects with two or more symptoms for ≥12 weeks, with one symptom being nasal blockage/obstruction or rhinorrhoea. Other symptoms could be facial pain or reduction/loss of smell. Subjects with asthma were defined as those with physician-diagnosed asthma, with at least one current symptom of; wheeze, trouble breathing, or sudden breathlessness or use of asthma medication. Those grouped into asthma only, did not meet the EPOS2012 criteria for CRS. Subjects in the asthma + CRS group fulfilled both the criteria for asthma and CRS.

All subject included had withdrawn from antihistamines for 72 h, long acting beta agonist (LABA) for 24 h and short acting beta agonist (SABA) for 8 h and Spiriva for 24 h prior to sample collection.

Both study I and study II were approved by the Regional Ethical Approval Committee in Gothenburg, Sweden (no. 593-08) with written informed consent given by all participants.

### Collection of nasal lavage and isolation of exosomes

NLF was collected as previously described [2]. Briefly, 5 ml of saline was instilled in the left nostril of the subjects, while tilting their head back. NLF was collected by passive dripping of the fluid into a container when subjects tilted their head forward. This procedure was repeated for the right nostril, before the samples were centrifuged for 10 min at 300×*g* at 4 °C to pellet the cells. The supernatant was transferred to new tubes and stored at −80 °C. For study I, NLF was collected at multiple occasions from each participant, while for study II, NLF was collected only once per participant. Pool A and B for the LC–MS/MS experiment in study I consisted of 70 ml NLF each, while the pools for study II consisted of 20–30 ml NLF each. Exosome isolation was performed as previously described [2]. Briefly, NLF was thawed and transferred to ultracentrifuge tubes, with the remaining tube volume filled with PBS, before centrifuged at 16,500×*g* for 20 min at 4 °C to remove cells, cell debris and larger extracellular vesicles. The supernatant was filtered through a 0.2 µm filter (Starstedt, Nümbrecht-Rommelsdorf, Germany) before exosomes were pelleted by ultracentrifugation at 120,000×g for 70 min at 4 °C (Ti70 or Ti45 fixed angle rotors in a Optima L-90K Ultracentrifuge, Beckman Coulter, Bromma, Sweden).

### Immune cell isolation and migration assay

Human monocytes, natural killer cells (NK cells) and neutrophils were isolated from blood collected from healthy donors in EDTA tubes. For NK cells and monocytes peripheral mononuclear cells were first isolated using Leucosep^®^ Tubes (Greiner Bio-One GmbH, Frickenhausen, Germany) and ficoll according to the manufacturer’s protocol. Monocytes and NK cells were then isolated from the peripheral mononuclear cells using the Monocyte Isolation Kit II and NK Cell Isolation Kit (Miltenyi GmbH, Bergisch Glagbach, Germany) according to the manufacturer’s protocol. The purity of the isolated monocytes were determined by the detection of CD14 (BD Bioscience) by a FACSAria. The purity of the isolated NK cells were determined by a FACSAria and the detection of CD56 and CD16 and the absence of CD3 (BD Bioscience). Neutrophils were isolated directly from blood using the MACSxpress^®^ Neutrophil Isolation Kit (Miltenyi GmbH)) and purity was determined by May Grünwald—Gimsa stain and morphological inspection using a microscope. All cells were seeded in IMDM or RPMI-1640 supplemented with 100 units/ml penicillin, 100 µg/ml streptomycin and 110 µg/ml sodium pyruvate (Sigma-Aldrich).

The isolated monocytes (35,000 cells per well) were seeded to adhere onto the membrane of the lower chamber of a Boyden chemotaxis chamber (Neuro Probe Inc., Gaithersburg, MD, USA) in inverted orientation for 4 h. The assembly was inverted back to normal orientation prior to the start of the assay. NK cells (250,000 cells per well) and neutrophils (170,000 cells per well) were seeded in the upper chamber of a Boyden chemotaxis chamber. Thirty microliters of different dosages of NLF-derived exosomes (0.3–100 µg/ml) in supplemented media were added to the upper chamber for monocytes and to the lower chamber for NK cells and neutrophils. A 0.1 % gelatin coated 8 μm (monocytes and neutrophils) or a 5 µm (NK cells) pore size membrane filter was placed between the upper and the lower chamber (Neuro Probe, Gaithersburg, MD). The cells were then incubated for 12 h (monocytes and NK cells) or 5 h (neutrophils) in a 37 °C humidified incubator with 5 % CO2. For monocytes, the cells that had migrated towards the upper chamber membrane filter side were analysed. These cells were fixed in methanol for 10 min, stained with Giemsa and examined with a microscope (Zeiss Axioplan 2, Carl Zeiss, Jena, Germany). Data for each treatment are shown as cells per field of view and compared with untreated wells. For NK cells and neutrophils analysis of migrated cells was performed on the cells that had migrated into the lower chamber media. These cells were counted with a Bürker chamber and Trypan blue. Data for each treatment are shown as cells per µl and compared with untreated wells.

### Protein extraction and digestion

Proteins were extracted using lysis buffer [8 M urea, 4 % CHAPS, 0.2 % SDS, 1 mM EDTA, 50 mM triethylammonium bicarbonate (TEAB, pH 8.5)] and sonication in a water bath. Study I exosomes were extracted in 100 µl lysis buffer and sonicated for 3 × 20 s with 20 s rest between bursts, while study II exosomes were extracted in 50 µl lysis buffer and sonicated 3 × 5 min with 1 min rest between bursts. The samples were vortexed briefly and centrifuged before the protein concentration of each sample was determined using the Pierce 660 nm Protein Assay Reagent (Thermo Fisher Scientific Inc., Waltham, MA, USA). Study I pool A protein (87 µg), study I pool B protein (68 µg) and study II pools (35 µg each) were diluted fourfold to give a final concentration of 250 mM TEAB (pH 8.5), 2 M urea, 1 % CHAPS, 0.05 % SDS and 0.25 mM EDTA. A reference pool sample for study II was made by pooling aliquots of the samples with equal amounts of each health/disease represented. The refrence pool was treated and diluted identically to the samples. The samples were reduced with 2 µl 50 mM tris(2-carboxyethyl)phosphine) (TCEP) and incubated for 60 min at 37 °C) (study I) or incubated, shaking, at room temperature for 40 min before incubated at 37 °C for 40 min (study II). The samples were then alkylated in 1 µl 200 mM methyl methanethiolsulfonate (MMTS) with 20 min incubation at room temperature. Digestion of the proteins was achieved by adding 50 µl milli-Q water to 20 µg trypsin (Promega Corporation, WI, USA), with 10 µl trypsin added to each sample and incubated overnight at 37 °C.

### Tandem mass tags labelling of peptides (study II)

Digested peptides from each sample in study II were labelled with reagents from the TMTsixplex Label Reagent Set (Pierce through Thermo Fisher Scientific) according to manufacturer’s instructions. Each sample in the set, consisting of one pooled reference sample and the pooled subject samples, was labelled with the TMT reagents. The reference pool, control, asthma + CRS and asthma only samples were labelled with reagents TMT6-126, TMT6-127, TMT6-128 and TMT6-129 respectively.

### Strong cation exchange fractionation of peptides

Strong cation exchange (SCX) chromatography was used to remove unbound TMT reagents (study II) and reduce the sample complexity by fractionation (study I and II). The concentrated peptides were acidified with 10 % formic acid (FA) and diluted with SCX solvent A [25 mM ammonium formate, pH 2.8, 25 % acetonitrile (ACN)] and injected onto a PolySULFOETHYL A SCX column (2.1 mm i.d. × 10 cm length, 5 μm particle size, 300 Å pore size). SCX chromatography and fractionation was carried out on an ÄKTA purifier system (GE Healthcare, Waukesha, WI, USA) at 0.25 mL/min flow rate using the following gradient: 0 % B (500 mM ammonium formate, pH 2.8, 25 % ACN) for 5 min; 0–40 % B for 20 min; 40–100 % B for 10 min and 100 % B held for 10 min. UV absorbance at 254 and 280 nm was monitored while fractions were collected at 0.5 mL intervals and the volume reduced in a SpeedVac. The peptide containing fractions were combined with their adjacent fractions (two or three together) resulting in eight and nine fractions for study I pool A and B respectively and 10 fractions in study II samples. Combined SCX fractions were desalted on PepClean C18 spin columns according to manufacturer’s instructions (Thermo Fisher Scientific, Inc., Waltham, MA, USA).

### NanoLC-MS/MS analysis on LTQ-Orbitrap Velos instrument

Study I: The desalted and dried fractions were reconstituted into 0.1 % FA and analysed on a LTQ-Orbitrap Velos (Thermo Fisher Scientific) interfaced with an in-house constructed nano-LC column [11]. Two microliter sample injections were made with an Easy-nLC autosampler (Thermo Fisher Scientific), running at 200 nL/min. The peptides were trapped on a pre-column (45 × 0.075 mm i.d.) and separated on a reversed phase column, 200 × 0.075 mm, packed with 3 μm Reprosil-Pur C18-AQ particles. The gradient was as followed; 0–60 min 7–37 % ACN, 0.2 % FA, up to 80 % ACN, 0.2 % FA over 7 min and the last 3 min at 80 % ACN, 0.1 % FA.

LTQ-Orbitrap Velos settings were: spray voltage 1.6 kV, 1 microscan for MS1 scans at 60000 resolution (m/z 400), full MS mass range m/z 400–1800. The LTQ-Orbitrap Velos was operated in a data-dependent mode, with one MS1 FTMS precursor ion scan followed by collision induced dissociation (CID) MS2 scans of the ten most abundant doubly or multiply protonated ions in each FTMS scan. MS2 were collected with 1 microscans and a collision energy of 35 %. Dynamic exclusion within 20 ppm for 30 s was used after two repeats of MS2 of a precursor ion.

Exclusion lists were applied to more thoroughly investigate the proteome of healthy exosomes. Exclusion lists were prepared using the data base search results and compiling a list of m/z for all spectra matches that passed the criteria for identification within a 3 min retention window and five decimals precision. A second 2 µl injection of each sample was analysed with the application of an exclusion list, with the peptides identified in the first acquisition of each fraction being excluded from the MS2 analysis. A third sample was also analysed with peptides identified in the second acquisition per fraction excluded from MS2 analysis.

Study II: The analysis parameters and instrument settings for study II were as above for study I, with the following modifications: The gradient was as follows: 0–60 min 5–25 % ACN, 60–70 min 25–40 % ACN, up to 80 % ACN over 10 min and held at 80 % ACN for 10 min. The LTQ-Orbitrap Velos was operated in a data-dependent mode with the top ten precursors from each MS1 scan selected for MS2 with high energy collision dissociation (HCD). The MS2 settings were as follows: collision energy of 40 %.

### Database search for protein identification (study I) and TMT quantitation (study II)

Study I: All MS raw data files per sample were merged for protein identification using Proteome Discoverer version 1.3 (Thermo Fisher Scientific). The database search was performed by Mascot search engine using the following criteria: *Homo sapiens* in SwissProt protein database (January 2012 FASTA db: SwissProt_2011_11.fasta. Version: 2.3 The number of human protein sequences were 20252 in the Swissprot database version November 2011.), MS peptide tolerance as 5 ppm, MS/MS tolerance as 0.5 Da, trypsin digestion allowing one missed cleavages with variable modifications; methionine oxidation and cysteine methylthiolation. The detected protein threshold was set to a false discovery rate (FDR) of 1 % confidence on peptide level (which resulted in a mascot significance threshold of 0.009) and identified proteins were grouped by sharing the same sequences to minimise redundancy. A final database search was performed to establish a nasal exosome proteome, using the same criteria as above, on a combination list of all three runs from both pools.

Study II: The MS raw data files from all fractions for one set were merged for relative quantification and identification using Proteome Discoverer 1.3 (Thermo Fisher Scientific). The database search was performed as above for study I with additional fixed modifications of lysine TMT6plex and N-terminal TMT6plex. For TMT quantification, the ratios of the TMT reporter ion intensities in the MS/MS spectra (126.12–130.14) from raw datasets were used to calculate fold changes between samples. Ratios were derived from Proteome Discoverer version 1.3 using the following criteria: 80 ppm mass tolerance of the reporter ions and calculating the intensity for the centroid peak, TMT reagent purity correction factors were used stated in the product data sheet thus recalculating the ratio depending on the reporter purity and adjacent reporters, the minimum ion intensity of a reporter was set to a threshold of 200 and missing values for reporter ions were replaced with the minimum intensity of the noise level in the spectra. Only unique peptides for a protein were considered for relative quantitation. Proteome Discoverer normalized the channel ratios, for each channel, normalized all peptide ratios by the median peptide ratio. The median protein ratio should be 1. The normalized ratios were exported into Excel for manual data interpretation. The detected protein threshold was set to a FDR of 1 % confidence on peptide level (which resulted in a mascot significance threshold of 0.0241).

### Bioinformatics analysis of proteomic data

Identified and quantified proteins were further analysed using Ingenuity Pathways Analysis (IPA) (Ingenuity^®^ Systems, http://www.ingenuity.com) to determine the most strongly associated functions and pathways. The dataset, containing gene accession numbers and corresponding expression values, was uploaded to IPA [(version 11904312, 12402621 (study I) and 14400082 (study II)]. Each identifier, where possible, was mapped to the corresponding object in the Ingenuity Pathways Analysis Knowledge Base (IPAKB). The fold changes for study II were calculated by dividing the two disease samples (*Asthma only* and *Asthma* + *CRS*) of pool A by the *control* of pool A and the disease samples of pool B by the control of pool B for comparisons of interest. Furthermore, the disease samples were compared to each other within each pool to find disease specific differences. The functional analysis of the dataset identified the biological functions and/or diseases most significant to the dataset. Molecules from the study II dataset that met the fold change cut-off of 1.3 were considered for analysis. A right‐tailed Fisher’s exact test was used to calculate a P value, determining the probability that each biological function and/or disease assigned to that dataset is due to chance alone. A Benjamini-Hochberg multiple testing correction was also applied.

The Gene Ontology (GO) is an initiative to describe biological functions, molecular processes and cellular components of genes. An open source software, GO Term Finder (http://www.go.princeton.edu/cgi-bin/GOTermFinder) [12], was used to analyse the enriched GO terms of the nasal exosome proteome and draw conclusions about the statistical significance of each gene ontology term compared to the genomic frequency. The annotation of GOA + Ensembl Xrefs—H. Sapiens (human) was used to determine the enriched terms. An interactive Venn diagram tool, VENNY (http://www.bioinfogp.cnb.csic.es/tools/venny/index.html), was used for comparing lists of proteins [13]. Information from the exosome database EVpedia (http://www.evpedia.info/) [14] were accessed July 2015.

Nine published exosomal proteome were used for analyses and included particularly relevant proteomes of healthy body fluid exosomes similar to nasal exosomes, i.e. from a mixed cellular origin, such as breast milk [15], semen [16], plasma [17], parotid saliva [18], saliva [19] and two sets from urine (Urine 1 http://www.dir.nhlbi.nih.gov/papers/lkem/exosome/) [20, 21] and Urine 2 [22]), as well as exosomes from two primary cell cultures (trabecular meshwork cells from the eye [23] and tracheobronchial ciliated epithelial cells [24]).

### Protein measurement for validation experiments

Exosomes were isolated as described above and resuspended in PBS. The protein concentration was determined by Pierce^®^ BCA Protein Assay Kit according to the manufacturer’s instructions (Thermo Fisher Scientific).

### Validation of mass spectrometry-identified proteins by flow cytometry

Exosomes (20 µg/40,000 beads) were incubated with anti-MHC class II-coated beads (custom-made by Dynal, Oslo, Norway, and kindly provided by S. Gabrielsson, Karolinska Institute, Stockholm, Sweden) or anti-CD63-coated beads (Life Technologies, Carlsbad, CA, USA) overnight at 4 °C with gentle agitation. The exosome-bead complexes were processed as described earlier [2, 25]. Briefly, the exosome-bead complexes were incubated with human IgG for 15 min at 4 °C, washed twice, before incubated with anti-CD9 (clone M-L13), anti-CD14 (clone MφP9), anti-CD63 (clone H5C6) antibodies or the appropriate isotype control for 40 min (all antibodies were from BD Bioscience, San Jose, CA, USA). The exosome-bead complexes were washed twice before analysed with FACSAria (BD Bioscience) and FlowJo software (Tri Star Inc, Ashland, OR, USA).

### Validation of mass spectrometry-identified proteins by Western Blot

Exosomes were isolated as described above and dissolved in 20 mM TrisHCl with 1 % SDS. As a control, monocyte-derived macrophages from buffy coat were used. To lyse the exosomes and the cells, the samples were freeze/thawed once at −20 °C and then sonicated twice for 5 min with vortexing between bursts. The samples were centrifuged at 13,000×*g* for 5 min and the supernatant were used for further analysis. Proteins (20 µg/well) were loaded and separated on NuPAGE^®^ Novex 4−12 % Bis–Tris gels (Invitrogen through Life Technologies) and transferred onto nitrocellulose membranes (Invitrogen through Life Technologies) according to the manufacturer’s instructions, except for the transfer buffer, which was modified to 5 % methanol and 0.01 % SDS. The membranes were blocked overnight with 0.5 % Blotting Grade Blocker Non-Fat Dry Milk (Bio-Rad Laboratories, Hercules, CS, USA) in TBS, before being washed three times. For all washes, TBS-Tween (TBST) was used. The membranes were then incubated with either mouse-anti-human iNOS (1:1000; clone 2D2-B2; R&D Systems, Minneapolis, MN, USA), mouse-anti-human S100A8 (1:250; clone #749916; R&D Systems), mouse-anti-human TSG101 (1:1000; clone 4A10; Abcam, Cambridge, U.K.) or rabbit-anti-human calnexin (1:1000; clone H-70; Santa Cruz Biotechnology, Santa Cruz, CA, USA) diluted in 0.25 % non-fat dry milk in TBST for 2 h. The membranes were washed three times before incubation with the secondary antibody for 1 h. Secondary antibodies used were; donkey-anti-rabbit (1:10,000, Amersham through GE Healthcare) and sheep-anti-mouse (1:2000, Amersham through GE Healthcare), diluted in 0.25 % non-fat dry milk in TBST. The membranes were then analysed with the Amersham ECL Plus Western Blotting Detection System (GE Healthcare) and a VersaDoc 4000 MP (Bio-Rad Laboratories).

### NOS activity assay

The enzymatic activity of nitric oxide synthase (NOS) was measured with the Ultra Sensitive Assay for Nitric Oxide Synthase (Oxford Biomedical Research, Rochester Hills, MI, USA), according to manufacturer’s instructions. Shortly, the exosomes were lysed (sonication 2 × 3 min) before incubated with reaction buffer, NADPH part A, NADPH part B and NOS cofactors for 6 h at 37 °C and placed on ice for 5 min. The control samples were kept at −20 °C until this point. Nitrate reductase was added to both samples and control samples and incubated at room temperature for 20 min. The samples were centrifuged at 12,500 rpm, for 5 min at 4 °C and the supernatant were transferred to a 96 well plate in duplicates. Colour reagent 1 and 2 was added and the samples were vortexed for 5 min before being analysed at 540 nm.

### Statistical analysis

Clinical measurements were analysed using the Mann–Whitney U test and cell migration were analysed using Kruskal–Wallis test followed by Dunn’s multiple comparisons test in GraphPad Prism 6 (GraphPad Software Inc., La Jolla, CA, USA) to determine significant differences.

## Results and discussion

### Nasal lavage-derived exosomes induce cell migration in primary immune cells

**Fig. 2:**
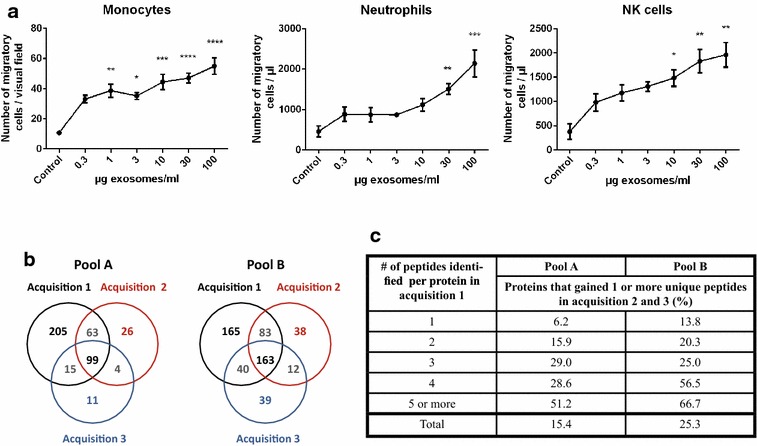
Nasal exosomes induce immune cell migration and the use of exclusion lists during mass spectrometry identifies new proteins and peptides. **a** Human monocytes, NK cells and neutrophils isolated from blood were added to one of the chambers of a Boyden chamber (35,000–250,000 cells/well). To the other chamber 30 µl of the different doses of nasal exosomes were added. Media was used as a control. After five (neutrophils) or 12 (monocytes and NK cells) hours the number of cells migrated to the exosome-containing chamber on the other side of the membrane were analysed. Kruskal–Wallis test followed by Dunn’s multiple comparisons test were used to determine significant differences where all concentrations were only compared to the control. P values * <0.05, ** <0.01, *** <0.001, **** <0.0001. **b** The Venn diagrams compare the proteins identified in the first acquisition (*black font*), the second acquisition (*red font*) and the third acquisition (*blue font*) and shows that the utilisation of exclusion lists in re-acquisitions led to increased numbers of identified proteins. **c** The utilisation of exclusion lists also resulted in the identification of new unique peptides for proteins previously identified, which increased the coverage and confidence for these proteins. Proteins were divided into groups based on the number of peptides identified in the first acquisition for each protein (1–5 or more). Data are presented as the percentage of proteins identified with additional unique peptides in the second or third acquisition in each category

We have previously reported on the presence of exosomes in the upper airways by analysing nasal lavage fluid (NLF) [2]. However, the possible biological functions of these nasal-derived exosomes have yet to be determined. Therefore, exosomes were isolated from NLF of healthy subjects, who showed no signs of decreased lung function or active inflammation, as determined by forced expiratory volume in 1 s (FEV1) and fractional exhaled nitric oxide (FeNO) respectively (Table 1, study I). To determine whether nasal exosomes can participate in innate immunity and induce immune cell trafficking, a cell migration assay was performed. Monocytes, NK cells and neutrophils isolated from blood were added to one of the chambers of a Boyden chemotaxis chamber, and different doses of nasal exosomes were added to the other chamber, with the number of cells migrating into the exosome-containing chamber determined after 5 or 12 h of incubation. Figure 2a shows that nasal exosomes induce a significant and dose-dependent migration of primary monocytes, NK cells and neutrophils. This suggests that nasal exosomes have the ability to participate in cell communication of the immune cells in the upper airways, complementing what has previously been shown for BALF exosomes in the lower airways [3, 26].

**Table 1:** Clinical characteristics of subjects participating in study I and II

	Study I	Study II		
	Healthy (n = 5)	Control^a^ (n = 14)	Asthma + CRS (n = 15)	Asthma only (n = 13)
Sex (M/F)	0/5	5/9	4/11	3/10
Age (years)	41 ± 7	40 ± 3	38 ± 3	44 ± 3
Body mass index	25.2 ± 1.2	24.4 ± 0.5	25.5 ± 0.9	27.4 ± 1.4
FEV1 (% predicted)	111 ± 6	101 ± 4	96 ± 4	93 ± 4
FeNO (ppb)	12 ± 1	15 ± 2	15 ± 5	19 ± 3
SPT (neg/pos)	3/1^b^	10/3^b^	4/11	2/11

Where appropriate, data are expressed as mean ± SEM

All subject included had withdrawn from antihistamines for 72 h, long acting beta agonist (LABA) for 24 h and short acting beta agonist (SABA) for 8 h and Spiriva for 24 h prior to sample collection

*M* male; *F* female; *FEV*_*1*_ forced expiratory volume in 1 s; *FeNO* fractional exhaled nitric oxide; *SPT* skin prick test

^a^The subjects in study I are also included in study II as controls

^b^One value missing

### Identification of nasal exosomal proteins by exclusion list-based LC–MS/MS

To determine whether the migration-inducing effects of nasal exosomes are related to their protein cargo, a detailed mass spectrometry approach was utilised. Exosomal protein was isolated from two pools of NLF and each sample was analysed using exclusion list based LC–MS/MS in order to thoroughly interrogate the proteome [27, 28]. After each acquisition, exclusion lists were constructed to exclude the peptides previously identified from the subsequent LC–MS/MS analysis, with each sample being analysed three times in total using two exclusion lists.

Analysis of the exosomal proteins from pool A identified 382 proteins in the first acquisition, with the second and third acquisitions identifying 30 and 11 additional proteins respectively. Analysis of the exosomal protein from pool B identified 451 proteins in the first acquisition, while the second and third acquisitions identified 50 and 39 new proteins respectively. In total, the two exclusion lists applied for pool A and B identified 11 and 20 % new proteins respectively compared to the first LC–MS/MS acquisition alone, showing that additional proteins can be found by the exclusion list approach (Fig. 2b). Important exosomal proteins such as annexin A1, rab-14, 14-3-3 protein epsilon, LAMP and heat shock protein HSP 90-alpha, were among the new proteins identified in the second and third acquisitions, demonstrating that exclusion lists can assist in a more thorough analysis of the exosomal proteome. Furthermore, the application of exclusion lists also increased the coverage of several proteins, by increasing the number of unique peptides identified for proteins found in the first acquisition. In total, 173 proteins in pool A and B gained one or more unique peptides by using exclusion lists (Fig. 2c). This increased coverage is especially valuable for the proteins identified with a single peptide in the first acquisition which can be confirmed by additional unique peptides in the additional acquisitions. Thus, the application of exclusion lists to proteomic analyses of exosomes can result in a more detailed description of proteomes, which is important for the understanding of exosomal biogenesis and uptake mechanisms and ultimately, for predicting exosomal protein-associated functions in health and various disorders.

The spectra from all acquisitions, from both pools, were combined to perform a new database search. This search identified 604 proteins which were used for downstream analyses (Additional file 1: Table S1). Of the 604 proteins, 289 were identified with two or more peptides and 293 were identified in both pools (Additional file 1: Table S1, “Identified in both pools”). To confirm the presence of exosomes in the nasal lavage isolates, the identified proteome was compared with a proposed “core exosome proteome” of 143 proteins [29], of which 82 (57 %) were identified in the nasal exosomal proteome (Additional file 1: Table S1, “Core exosome proteome”). The proteomic detection of previously identified exosomal proteins supports the validity of the exosome isolation method, as well as the sensitivity of the proteomic approach applied in the present study.

### The nasal exosome proteome is specifically associated with immune-related functions

**Fig. 3:**
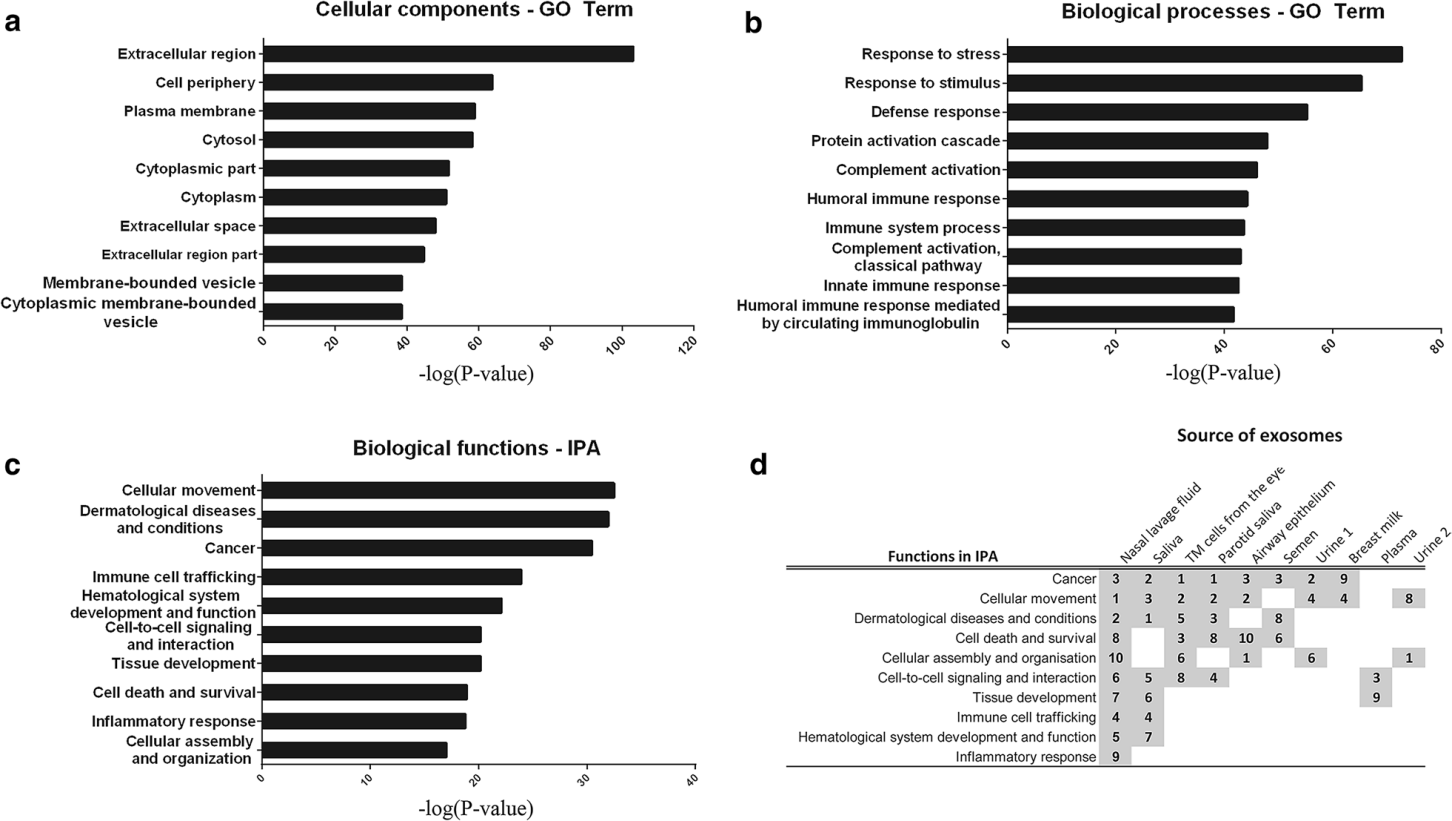
Immune-related functions are specifically associated with nasal exosomal. GO Term Finder was used to determine the most enriched cellular components (**a**) and biological processes (**b**) in the nasal exosomal proteome of healthy subjects, compared to the genome frequency. The 10 most enriched terms (based on *P* value) in each category are displayed. **c** IPA was used to determine the most associated biological functions with the nasal exosomal proteome. The 10 most associated biological functions (based on P value) are displayed. **d** The proteomes of nine previously published exosomal studies were analysed with IPA to determine the specificity of the functions associated with the nasal exosomal proteome. The top 10 ranked functions associated with nasal lavage exosomes were compared to the rank attained in the other exosomal proteomes. Numbers represent the rank (1–10) for a particular function within each exosomal proteome. The nine exosomal proteomes used for the comparison were derived from; saliva [19], trabecular meshwork (TM) cells from the eye [23], parotid saliva [18], tracheobronchial ciliated epithelial cells [24], semen [16], urine [20–22], breast milk [15] and plasma [17]

The 604 proteins of the nasal exosome proteome were analysed using GO Term Finder to identify the most enriched GO terms and the associated proteins. The cellular components most enriched in nasal exosomes were related to parts of *the extracellular region*, *the plasma membrane* and *the cytoplasmic region* (Fig. 3a), but nasal exosomes also contained 94 proteins associated with the GO term *membrane*-*bound vesicles* (Additional file 1: Table S1, **“**Membrane-bound vesicle proteins”), which has exosomes as a subgroup.

Importantly, biological processes enriched in the nasal exosomal proteome included *defense response* and *immune system processes* (Fig. 3b) and more specifically, 80 proteins categorised as *innate immune response*, including S100 proteins, inducible nitric oxide synthase (NOS2) and BPIF proteins, supporting an immune-related function for nasal exosomes.

Ingenuity pathway analysis (IPA) was utilised to further analyse the possible functions associated with the nasal exosome proteome, revealing associations with biological functions such as *cellular movement*, *dermatological diseases and conditions* and *cancer*, as well as *immune cell trafficking* and *inflammatory response* (Fig. 3c), again connecting the nasal exosomal proteome to immune-related functions. To determine the specificity of the identified associated functions, the datasets of nine published exosome proteome from relevant, healthy, human in vivo sources were also analysed using IPA and compared to the nasal exosomal IPA analysis. The comparison of these studies to the nasal exosome proteome showed that the biological functions of *cellular movement*, *dermatological diseases and conditions* and *cancer* are common to multiple studies and are often the top functions (Fig. 3d). By contrast, *immune cell trafficking*, *inflammatory response* and *hematological system development and function* were only the top functions in one of the nine studies analysed. Of the 604 proteins in the nasal exosome, 205 proteins belonged to one or more of these specifically associated functions (Additional file 1: Table S1, **“**Immune-related proteins”). Specific annotations for *immune cell trafficking* and *inflammatory response* included migration, movement and adhesion of various immune cells, including leukocytes, phagocytes and myeloid cells. Thus, compared to other exosomal proteomes, the nasal exosome proteome is particularly associated with the immune-regulatory functions.

The important comparative analyses of the nasal exosome proteome to other proteomes enable a better understanding of the exosomes and their potential function. These analyses are limited by the datasets deposited into the public repositories and are only possible if the proteomic datasets are made public by submission to proteomic repositories or to the growing number of exosomal proteomic databases. Our study highlights the importance of submitting proteome lists to public databases, which have enabled the specific features of nasal exosomes to be identified.

### Validation of proteins identified by mass spectrometry

**Fig. 4:**
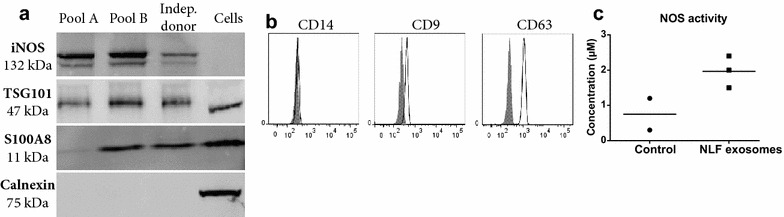
Validation of mass spectrometry identified proteins using Western blot and flow cytometry. **a** The presence of iNOS, TSG101 and S100A8 were validated with Western blot in pool A and pool B of healthy subjects and a fifth subject (“indep. donor”). The absence of calnexin in exosomes was also demonstrated. **b** CD14, CD9 and CD63 were validated by binding the exosomes to anti-MHC class II beads and analysis with flow cytometry.* Grey filled* curve shows isotype control and* black open curve* shows CD14, CD9 and CD63 respectively. **c** The iNOS enzyme was demonstrated to be biochemically functional by an activity assay. Nitric oxide synthase coverts l-arginine into nitric oxide, which is further converted to nitrite. The assay measures the concentration of nitrite (µM), shown on the* y-axis*. Control samples (“Ctrl”) show the baseline concentration of nitrite in nasal exosomes. Nasal lavage exosomes (“NLF exo”) samples shows a higher concentration of nitrite after the assay

Several proteins identified by mass spectrometry were validated using Western blot or flow cytometry. Proteins were selected for validation if they were common to exosomes (CD63, CD9 and TSG101), unique to nasal exosomes or immune-related (NOS2 and S100A8) or potentially indicative of the cellular origin of nasal exosomes (the monocyte/macrophage marker, CD14). Western blotting confirmed the presence of TSG101, NOS2 and S100A8 in exosomes isolated from pools A and B, as well as in exosomes of an independent healthy subject. Calnexin, an endoplasmic reticulum protein, was not identified using mass spectrometry and its absence was validated using Western blot (Fig. 4a).

Flow cytometry confirmed the direct presence of CD63 and CD9 and the indirect presence of MHC class II with the use of anti-MHC class II coated beads. The nasal exosomes also had minor positivity for CD14, which suggests that at least a small part of the isolated exosomes may originate from monocytes or macrophages (Fig. 4b). Together, the flow cytometry and Western blot confirmed the presence of several of the proteins identified with LC–MS/MS by other techniques.

### Enzyme activity within nasal exosomes

The innate immune system includes nitric oxide (NO) as part of the first response against a bacterial infection, which is synthesised by a family of nitric oxide synthase enzymes (NOSs). NOS2 is constitutively expressed in the airway epithelium, but can be up-regulated during inflammation [30, 31]. NOS2 has not previously been identified in exosomes (exosomal proteome database, EVpedia [14] July, 2015) and the identification of NOS2 in nasal exosomes is therefore particularly interesting. As seen in Fig. 4a, its presence was confirmed in both pools of healthy exosomes by Western blot, but importantly, the biochemical activity of NOS2 was confirmed in an enzyme activity assay (Fig. 4c), showing that the protein is not only intact in nasal exosomes, but also biologically active.

### Altered expression of mucin-, serum-, barrier- and antimicrobial-associated proteins in exosomes from subjects with airway inflammation

A quantitative analysis was conducted to determine the influence of respiratory disease on the nasal exosome proteome. Exosomes were therefore isolated from three groups of subjects; *control*, *asthma only*, and *asthma* + *CRS* (clinical characteristics of the patients are shown in Table 1, study II). Two independent pools of NLF from *control*, *asthma only* and *asthma* + *CRS* groups were constructed for proteomic analysis (Fig. 1, study II). Of the total 179 proteins identified in the dataset, 140 proteins were quantified. Proteins quantified in all samples of study II (74 proteins) were analysed to examine proteins with consistent expression across both pools. Consistent expression changes of several proteins were observed in both pools of *asthma only* and *asthma* + *CRS* subjects compared to *controls.* Mucin-7 was increased in both disease groups compared to *controls* (Table 2a), with mucin-5B also increased in *asthma only* (Table 2c). Hypersecretion of mucin is one of the hallmark features of asthma [32], with both mucin-7 and mucin-5B being associated with asthma [33, 34]. Furthermore, haptoglobin and hemoglobin subunit beta were also increased in both the *asthma only* and *asthma* + *CRS* samples compared to *control* (Table 2a). This increase in serum-associated proteins was further supported by the increase of serum albumin in *asthma* + *CRS* compared to *control* (Table 2b). Vascular proteins have previously been identified in NLF [35], but an increase in these proteins compared to *control* may indicate epithelial damage and plasma exudation. The increase could also reflect the chronic inflammation associated with both asthma and CRS, as haptoglobin is an acute phase protein that is associated with inflammation [36].

**Table 2:** Proteins with altered expression in airway disease

Acc.	Protein	Asthma + CRS vs control		Asthma only vs control		Asthma + CRS vs Asthma only	
		A	B	A	B	A	B
A^a^							
Q8TAX7	MUC7	+4.0	+1.4	+2.9	+1.8		
P68871	HBB	+2.2	+1.3	+2.0	+2.7		
P00738	HP	+1.8	+1.3	+1.7	+1.8		
P02788	LTF	−1.5	−1.3	−1.9	−1.6		
Q9GZZ8	LACRT	−1.3	−2.0	−1.5	−2.8		
B^b^							
P02768	ALB	+1.3	+1.4				
P01857	IGHG1	+1.3	+1.6				
P05109	S100A8	−1.5	−1.5				
P06702	S100A9	−1.5	−1.4				
P80511	S100A12	−1.4	−2.3				
C^c^							
Q9HC84	MUC5B			+2.2	+2.4		
P20930	FLG			−1.4	−2.9		
P01591	IGJ			−1.3	−1.5		
P01876	IGHA1			−1.4	−1.3		
P01833	PIGR			−1.3	−1.4		
Q86YZ3	HRNR			−1.8	−3.8		
D^d^							
Q08380	LGALS3BP					+1.6	+1.5
Q86YZ3	HRNR					+3.6	+1.3
P05109	S100A8					−1.3	−2.1
P06702	S100A9					−1.3	−1.9
P80511	S100A12					−1.6	−1.8
P60709	ACTB					−1.2	−1.5
P35579	MYH9					−1.6	−1.6
P08311	CTSG					−2.9	−1.5

Only proteins with greater than 1.3-fold change in both pool A and pool B are presented. A negative value (“−”) means negative fold change of the first disease compared to second state, while a positive value (“+”) means positive fold change

*Acc* GenBank accession number; *ACTB* actin, cytoplasmic 1; *ALB* serum albumin; *CTSG* cathepsin G; *FLG* filaggrin; *HBB* hemoglobin subunit B; *HP* haptoglobin; *HRNR* hornerin; *IGHA1* Ig alpha-1 chain C region; *IGHG1* Ig gamma-1 chain C region; *IGJ* Immunoglobulin J chain; *LACRT* extracellular glycoprotein lacritin; *LGALS3BP* Galectin-3-binding protein; *LTF* lactotransferrin; *MUC5B* mucin-5B; *MUC7* mucin-7; *MYH9* myosin-9; *PIGR* polymeric immunoglobulin receptor

^a^Proteins altered in both *asthma* + *CRS* and *asthma only* against the *control*

^b^Proteins altered in *asthma* + *CRS* compared to *control*

^c^Proteins altered in *asthma only* compared to *control*

^d^Proteins altered in *asthma* + *CRS* compared to *asthma only*

A group of S100 proteins; S100A8, S100A9 and S100A12, were consistently decreased in NLF exosomes from *asthma* + *CRS* compared to *control* (Table 2b) and compared to *asthma only* (Table 2d). S100 proteins have been shown to be involved in antifungal and antibacterial activity [37], regulation of leukocyte adhesion and migration and promotion of cytokine and chemokine production [38], as well as the induction of pro-inflammatory responses in monocytes [39]. Furthermore, S100A8 and S100A9 positive exosomes were recently demonstrated to be chemotactic for immune cells [40]. The decreased expression of these proteins that we found here is consistent with the previous findings that S100 proteins are decreased in nasal epithelial cells and NLF from patients with CRS [41]. A decrease in the expression of S100 proteins could suggest impaired barrier function and increased susceptibility to bacterial and fungal overgrowth. The decreased expression of cathepsin G in the *asthma* + *CRS* group compared to *asthma only* may be further evidence for decreased antifungal response (Table 2d) as cathepsin G deficiency has previously been associated with increased susceptibility to fungal infections [42].

The *asthma only* group was found to have several exosomal proteins with consistently decreased expression compared to the *control* group, including filaggrin, hornerin and three immunoglobulin-related proteins (Table 2c). Both filaggrin and hornerin have been implicated in the barrier function of the skin, with mutations in these genes associated with barrier dysfunction, atopic dermatitis and increased asthma severity in children [43–46]. Asthma is an inflammatory airway disorder, however there is increasing evidence that the epithelium has an important role in the interaction with allergens [47] and disease progression [48]. The importance of hornerin and filaggrin in the barrier function of the airways requires further evaluation, but similar to skin, the airway epithelium is an important barrier of the host defence system.

## Conclusion

This study provides the first description of the proteome of nasal exosomes and suggests a potential role in upper airways disease. The application of exclusion lists increased both the confidence and number of proteins identified, with 604 proteins identified in the nasal exosomes of healthy individuals. Many of the identified proteins were associated with immune-related functions, which was specific for nasal exosomes compared to previously published exosomal proteomes. The current study is the first to use isobaric tags to quantify exosomal proteins in human disease and it revealed that several groups of proteins are consistently altered in nasal exosomes in subjects with *asthma* + *CRS* and *asthma only* compared to *control*. An increased expression in exosomal mucin and serum-associated proteins was observed in subjects with airway diseases, which may reflect the inflammatory processes. The decreased expression in barrier and antimicrobial proteins could possibly contribute to increased susceptibility to infections, which has important clinical implications in disease progression.

The migration of immune cells to the site of inflammation or infection is an important part of the innate immune system, especially in the nose, where a strong first line of defence is crucial. Interestingly, immune cell trafficking was associated with both the baseline healthy exosome dataset and with several of the proteins altered with disease. Most importantly, it was also demonstrated that nasal exosomes can induce migration in several immune cells, such as monocytes, NK cells and neutrophils in vitro. This suggests that nasal exosomes can participate in the recruitment of immune cells to the nose and furthermore, that the capability to do so may be altered during inflammatory airway diseases.

## Additional file

10.1186/s12967-016-0927-4 This table contains the list of the 604 proteins identified in healthy nasal exosomes. Information found in this table includes; number of peptides identified, the NLF-derived exosomal proteins included in; “core exosome proteome”, “membrane-bound vesicle proteins” and “immune-related proteins”.

## Abbreviations

ACN: acetonitrile
BALF: bronchoalveolar lavage fluid
CRS: chronic rhinosinusitis
FA: formic acid
FDR: false discovery rate
FeNO: exhaled nitric oxide
FEV1: forced expiratory volume in 1 s
GO: gene ontology
IPA: ingenuity pathways analysis
LC: liquid chromatography
MS: mass spectrometry
NK: natural killer
NLF: nasal lavage fluid
NO: nitric oxide
NOS: nitric oxide synthase
SABA: short acting beta agonist
SCX: strong cation exchange
TMT: tandem mass tag

## References

[1] Admyre C, Grunewald J, Thyberg J, Gripenback S, Tornling G, Eklund A (2003). Exosomes with major histocompatibility complex class II and co-stimulatory molecules are present in human BAL fluid. Eur Respir J.

[2] Lässer C, O’Neil SE, Ekerljung L, Ekström K, Sjöstrand M, Lötvall J (2011). RNA-containing exosomes in human nasal secretions. Am J Rhinol Allergy.

[3] Torregrosa Paredes P, Esser J, Admyre C, Nord M, Rahman QK, Lukic A (2012). Bronchoalveolar lavage fluid exosomes contribute to cytokine and leukotriene production in allergic asthma. Allergy.

[4] Levanen B, Bhakta NR, Paredes PT, Barbeau R, Hiltbrunner S, Pollack JL (2013). Altered microRNA profiles in bronchoalveolar lavage fluid exosomes in asthmatic patients. J Allergy Clin Immunol.

[5] Fokkens WJ, Scheeren RA (2000). Upper airway defence mechanisms. Paediatr Respir Rev.

[6] Lötvall J, Ekerljung L, Rönmark EP, Wennergren G, Linden A, Rönmark E (2009). West Sweden Asthma Study: prevalence trends over the last 18 years argues no recent increase in asthma. Respir Res.

[7] Hastan D, Fokkens WJ, Bachert C, Newson RB, Bislimovska J, Bockelbrink A (2011). Chronic rhinosinusitis in Europe—an underestimated disease. A GA(2)LEN study. Allergy.

[8] Eriksson J, Ekerljung L, Ronmark E, Dahlen B, Ahlstedt S, Dahlen SE (2012). Update of prevalence of self-reported allergic rhinitis and chronic nasal symptoms among adults in Sweden. Clin Respir J.

[9] Lötvall J, Ekerljung L, Lundbäck B (2010). Multi-symptom asthma is closely related to nasal blockage, rhinorrhea and symptoms of chronic rhinosinusitis-evidence from the West Sweden Asthma Study. Respir Res.

[10] Fokkens WJ, Lund VJ, Mullol J, Bachert C, Alobid I, Baroody F (2012). European position paper on rhinosinusitis and nasal polyps 2012. Rhinol Suppl.

[11] Carlsohn E, Nystrom J, Karlsson H, Svennerholm AM, Nilsson CL (2006). Characterization of the outer membrane protein profile from disease-related Helicobacter pylori isolates by subcellular fractionation and nano-LC FT-ICR MS analysis. J Proteome Res.

[12] Boyle EI, Weng S, Gollub J, Jin H, Botstein D, Cherry JM (2004). GO::TermFinder–open source software for accessing Gene Ontology information and finding significantly enriched Gene Ontology terms associated with a list of genes. Bioinformatics.

[13] VENNY. An interactive tool for comparing lists with Venn Diagrams. 2007. http://www.bioinfogp.cnb.csic.es/tools/venny/index.html.

[14] Kim DK, Lee J, Kim SR, Choi DS, Yoon YJ, Kim JH (2015). EVpedia: a community web portal for extracellular vesicles research. Bioinformatics.

[15] Admyre C, Johansson SM, Qazi KR, Filen JJ, Lahesmaa R, Norman M (2007). Exosomes with immune modulatory features are present in human breast milk. J Immunol.

[16] Poliakov A, Spilman M, Dokland T, Amling CL, Mobley JA (2009). Structural heterogeneity and protein composition of exosome-like vesicles (prostasomes) in human semen. Prostate.

[17] Looze C, Yui D, Leung L, Ingham M, Kaler M, Yao X (2009). Proteomic profiling of human plasma exosomes identifies PPARgamma as an exosome-associated protein. Biochem Biophys Res Commun.

[18] Gonzalez-Begne M, Lu B, Han X, Hagen FK, Hand AR, Melvin JE (2009). Proteomic analysis of human parotid gland exosomes by multidimensional protein identification technology (MudPIT). J Proteome Res.

[19] Ogawa Y, Miura Y, Harazono A, Kanai-Azuma M, Akimoto Y, Kawakami H (2011). Proteomic analysis of two types of exosomes in human whole saliva. Biol Pharm Bull.

[20] Pisitkun T, Shen RF, Knepper MA (2004). Identification and proteomic profiling of exosomes in human urine. Proc Natl Acad Sci USA.

[21] Gonzales PA, Pisitkun T, Hoffert JD, Tchapyjnikov D, Star RA, Kleta R (2009). Large-scale proteomics and phosphoproteomics of urinary exosomes. J Am Soc Nephrol.

[22] Wang Z, Hill S, Luther JM, Hachey DL, Schey KL (2012). Proteomic analysis of urine exosomes by multidimensional protein identification technology (MudPIT). Proteomics.

[23] Stamer WD, Hoffman EA, Luther JM, Hachey DL, Schey KL (2011). Protein profile of exosomes from trabecular meshwork cells. J Proteomics.

[24] Kesimer M, Scull M, Brighton B, DeMaria G, Burns K, O’Neal W (2009). Characterization of exosome-like vesicles released from human tracheobronchial ciliated epithelium: a possible role in innate defense. FASEB J.

[25] Lässer C, Alikhani VS, Ekström K, Eldh M, Paredes PT, Bossios A (2011). Human saliva, plasma and breast milk exosomes contain RNA: uptake by macrophages. J Transl Med.

[26] Qazi KR, Torregrosa Paredes P, Dahlberg B, Grunewald J, Eklund A, Gabrielsson S (2010). Proinflammatory exosomes in bronchoalveolar lavage fluid of patients with sarcoidosis. Thorax.

[27] Chen HS, Rejtar T, Andreev V, Moskovets E, Karger BL (2005). Enhanced characterization of complex proteomic samples using LC-MALDI MS/MS: exclusion of redundant peptides from MS/MS analysis in replicate runs. Anal Chem.

[28] Muntel J, Hecker M, Becher D (2012). An exclusion list based label-free proteome quantification approach using an LTQ Orbitrap. Rapid Commun Mass Spectrom.

[29] Mathivanan S, Ji H, Simpson RJ (2010). Exosomes: extracellular organelles important in intercellular communication. J Proteomics.

[30] Asano K, Chee CB, Gaston B, Lilly CM, Gerard C, Drazen JM (1994). Constitutive and inducible nitric oxide synthase gene expression, regulation, and activity in human lung epithelial cells. Proc Natl Acad Sci USA.

[31] Yan ZQ, Hansson GK, Skoogh BE, Lotvall JO (1995). Induction of nitric oxide synthase in a model of allergic occupational asthma. Allergy.

[32] Evans CM, Koo JS (2009). Airway mucus: the good, the bad, the sticky. Pharmacol Ther.

[33] Rousseau K, Vinall LE, Butterworth SL, Hardy RJ, Holloway J, Wadsworth ME (2006). MUC7 haplotype analysis: results from a longitudinal birth cohort support protective effect of the MUC7*5 allele on respiratory function. Ann Hum Genet.

[34] Kirkham S, Sheehan JK, Knight D, Richardson PS, Thornton DJ (2002). Heterogeneity of airways mucus: variations in the amounts and glycoforms of the major oligomeric mucins MUC5AC and MUC5B. Biochem J.

[35] Casado B, Pannell LK, Iadarola P, Baraniuk JN (2005). Identification of human nasal mucous proteins using proteomics. Proteomics.

[36] Gabay C, Kushner I (1999). Acute-phase proteins and other systemic responses to inflammation. N Engl J Med.

[37] Mambula SS, Simons ER, Hastey R, Selsted ME, Levitz SM (2000). Human neutrophil-mediated nonoxidative antifungal activity against Cryptococcus neoformans. Infect Immun.

[38] Ryckman C, Vandal K, Rouleau P, Talbot M, Tessier PA (2003). Proinflammatory activities of S100: proteins S100A8, S100A9 and S100A8/A9 induce neutrophil chemotaxis and adhesion. J Immunol.

[39] Foell D, Wittkowski H, Kessel C, Luken A, Weinhage T, Varga G (2013). Proinflammatory S100A12 can activate human monocytes via Toll-like receptor 4. Am J Respir Crit Care Med.

[40] Burke M, Choksawangkarn W, Edwards N, Ostrand-Rosenberg S, Fenselau C (2014). Exosomes from myeloid-derived suppressor cells carry biologically active proteins. J Proteome Res.

[41] Tieu DD, Peters AT, Carter RG, Suh L, Conley DB, Chandra R (2010). Evidence for diminished levels of epithelial psoriasin and calprotectin in chronic rhinosinusitis. J Allergy Clin Immunol.

[42] Tkalcevic J, Novelli M, Phylactides M, Iredale JP, Segal AW, Roes J (2000). Impaired immunity and enhanced resistance to endotoxin in the absence of neutrophil elastase and cathepsin G. Immunity.

[43] Palmer CN, Irvine AD, Terron-Kwiatkowski A, Zhao Y, Liao H, Lee SP (2006). Common loss-of-function variants of the epidermal barrier protein filaggrin are a major predisposing factor for atopic dermatitis. Nat Genet.

[44] Henry J, Hsu CY, Haftek M, Nachat R, de Koning HD, Gardinal-Galera I (2011). Hornerin is a component of the epidermal cornified cell envelopes. FASEB J.

[45] Marenholz I, Nickel R, Ruschendorf F, Schulz F, Esparza-Gordillo J, Kerscher T (2006). Filaggrin loss-of-function mutations predispose to phenotypes involved in the atopic march. J Allergy Clin Immunol.

[46] Palmer CN, Ismail T, Lee SP, Terron-Kwiatkowski A, Zhao Y, Liao H (2007). Filaggrin null mutations are associated with increased asthma severity in children and young adults. J Allergy Clin Immunol.

[47] Joenvaara S, Mattila P, Renkonen J, Makitie A, Toppila-Salmi S, Lehtonen M (2009). Caveolar transport through nasal epithelium of birch pollen allergen Bet v 1 in allergic patients. J Allergy Clin Immunol..

[48] Holgate ST (2007). Epithelium dysfunction in asthma. J Allergy Clin Immunol.

