# Supplementary material for: Exosomes in the nose induce immune cell trafficking and harbour an altered protein cargo in chronic airway inflammation
Source: J Transl Med. 2016 Jun 20;14:181. doi: 10.1186/s12967-016-0927-4 (PMC4913423; doi:10.1186/s12967-016-0927-4)
Supplement: Supplementary file 1 — 10.1186/s12967-016-0927-4 This table contains the list of the 604 proteins identified in healthy nasal exosomes. Information found in this table includes; number of peptides identified, the NLF-derived exosomal proteins included in; “core exosome proteome”, “membrane-bound vesicle proteins” and “immune-related proteins”. [file 12967_2016_927_MOESM1_ESM.pdf]

Table S1.The list of the 604 proteins identified in nasal exosomes and the lists of proteins identifed with IPA and GO Term finder.

Accession #	Description	Gene name	# Peptides	Identified in both pools	Core exosome proteome	Membrane-bound vesicle proteins	Immune-related proteins
Q04446	1,4-alpha-glucan-branching enzyme	GBE1	1				
P31946	14-3-3 protein beta/alpha	YWHAB	3		X	X	
P62258	14-3-3 protein epsilon	YWHAE	1		X	X	
P31947	14-3-3 protein sigma	SFN	3	X	X		X
P27348	14-3-3 protein theta	YWHA	3				
P63104	14-3-3 protein zeta/delta	YWHAZ	4	X	X	X	
Q01970	1-phosphatidylinositol-4,5-bisphosphate phosphodiesterase beta-3	PLCB3	1	X			X
P09543	2',3'-cyclic-nucleotide 3'-phosphodiesterase	CNP	3	X	X	X	X
P62191	26S protease regulatory subunit 4	PSMC1	1				
P62266	40S ribosomal protein S23	RPS23	1	X			
P61247	40S ribosomal protein S3a	RPS3A	1				
Q9BRK5	45 kDa calcium-binding protein	SDF4	1	X			
P21589	5'-nucleotidase	NT5E	1				X
P62750	60S ribosomal protein L23a	RPL23A	1				
P39023	60S ribosomal protein L3	RPL3	1				
P46777	60S ribosomal protein L5	RPL5	1				
P18124	60S ribosomal protein L7	RPL7	1				
P52209	6-phosphogluconate dehydrogenase, decarboxylating	PGD	1	X			
O75173	A disintegrin and metalloproteinase with thrombospondin motifs 4	ADAMTS4	1				
Q8IZP0	Abl interactor 1	ABI1	1				X
Q13510	Acid ceramidase	ASAH1	1				
Q92485	Acid sphingomyelinase-like phosphodiesterase 3b	SMPDL3B	1				
P68032	Actin, alpha cardiac muscle 1	ACTC1	11				
P60709	Actin, cytoplasmic 1	ACTB	17	X	X		X
O15143	Actin-related protein 2/3 complex subunit 1B	ARPC1B	1				
O15144	Actin-related protein 2/3 complex subunit 2	ARPC2	2	X			

Accession #	Description	Gene name	# Peptides	Identified in both pools	Core exosome proteome	Membrane-bound vesicle proteins	Immune-related proteins
P59998	Actin-related protein 2/3 complex subunit 4	ARPC4	1				
P07108	Acyl-CoA-binding protein	DBI	2	X			
P23526	Adenosylhomocysteinase	AHCY	1		X	X	
Q01518	Adenylyl cyclase-associated protein 1	CAP1	4	X			
P61204	ADP-ribosylation factor 3	ARF3	1		X		
P43353	Aldehyde dehydrogenase family 3 member B1	ALDH3B1	5	X			
P30838	Aldehyde dehydrogenase, dimeric NADP-preferring	ALDH3A	4	X			
Q04828	Aldo-keto reductase family 1 member C1	AKR1C1	3				
P05186	Alkaline phosphatase, tissue-nonspecific isozyme	ALPL	7	X			X
P51993	Alpha-(1,3)-fucosyltransferase	FUT6	3	X			
P26572	Alpha-1,3-mannosyl-glycoprotein 2-beta-N-acetylglucosaminyltransferase	MGAT1	1				
P02763	Alpha-1-acid glycoprotein 1	ORM1	1				X
P01011	Alpha-1-antichymotrypsin	SERPINA3	2	X			X
P01009	Alpha-1-antitrypsin	SERPINA1	6	X		X	X
P04217	Alpha-1B-glycoprotein	A1BG	1				
P02765	Alpha-2-HS-glycoprotein	AHSG	1	X			X
P01023	Alpha-2-macroglobulin	A2M	26	X		X	X
A8K2U0	Alpha-2-macroglobulin-like protein 1	A2ML1	1				
P12814	Alpha-actinin-1	ACTN1	6	X		X	
O43707	Alpha-actinin-4	ACTN4	8	X	X	X	X
P06733	Alpha-enolase	ENO1	6	X	X		X
P54920	Alpha-soluble NSF attachment protein	NAPA	1				
Q12904	Aminoacyl tRNA synthase complex-interacting multifunctional protein 1	AIMP	1			X	X
Q9H4A4	Aminopeptidase B	RNPEP	1			X	
P15144	Aminopeptidase N	ANPEP	8	X	X	X	X
P04083	Annexin A1	ANXA1	5	X	X		X
P50995	Annexin A11	ANXA11	1	X	X	X	

Accession #	Description	Gene name	# Peptides	Identified in both pools	Core exosome proteome	Membrane-bound vesicle proteins	Immune-related proteins
Q5XXA6	Anoctamin-1	ANO1	6	X			
P03973	Antileukoproteinase	SLPI	7	X			X
P02647	Apolipoprotein A-I	APOA1	8	X		X	X
P02652	Apolipoprotein A-II	APOA2	2	X			X
P06727	Apolipoprotein A-IV	APOA4	4	X			X
P04114	Apolipoprotein B-100	APOB	5			X	X
P02654	Apolipoprotein C-I	APOC1	2	X			
P02649	Apolipoprotein E	APOE	1	X	X		X
P29972	Aquaporin-1	AQP1	1				X
P55064	Aquaporin-5	AQP5	1	X			
P16050	Arachidonate 15-lipoxygenase	ALOX15	3	X			X
P05089	Arginase-1	ARG1	1				X
P00505	Aspartate aminotransferase, mitochondrial	GOT2	1	X			
P53396	ATP-citrate synthase	ACLY	1		X		
Q96BJ3	Axin interactor, dorsalization-associated protein	AIDA	1	X			
P17213	Bactericidal permeability-increasing protein	BPI	1				X
Q8N4F0	Bactericidal/permeability-increasing protein-like 1	BPIL1	4	X			
P98160	Basement membrane-specific heparan sulfate proteoglycan core protein	HSPG2	30	X	X		X
O95395	Beta-1,3-galactosyl-O-glycosyl-glycoprotein beta-1,6-N-acetylglucosaminyltransferase 3	GCNT	2	X			X
Q8NES3	Beta-1,3-N-acetylglucosaminyltransferase lunatic fringe	LFNG	1				X
O60513	Beta-1,4-galactosyltransferase 4	B4GALT4	1				
P61769	Beta-2-microglobulin	B2M	5	X		X	X
Q562R1	Beta-actin-like protein 2	ACTBL2	5	X			
Q9Y5Z0	Beta-secretase 2	BACE2	1	X			
Q13867	Bleomycin hydrolase	BLMH	3	X			
Q10589	Bone marrow stromal antigen 2	BST2	1	X			X
Q9NP55	BPI fold-containing family A member 1	BPIFA1	1				X
Q8TDL5	BPI fold-containing family B member 1	BPIFB1	12	X			

Accession #	Description	Gene name	# Peptides	Identified in both pools	Core exosome proteome	Membrane-bound vesicle proteins	Immune-related proteins
P59826	BPI fold-containing family B member 3	BPIFB3	1	X			
P59827	BPI fold-containing family B member 4	BPIFB4	1	X			
P80723	Brain acid soluble protein 1	BASP1	2				
Q9UQB8	Brain-specific angiogenesis inhibitor 1-associated protein 2	BAIAP2	2				
P11274	Breakpoint cluster region protein	BCR	1				X
O75363	Breast carcinoma-amplified sequence 1	BCAS1	1				
P04003	C4b-binding protein alpha chain	C4BPA	4	X			X
P20851	C4b-binding protein beta chain	C4BPB	1				X
Q9H251	Cadherin-23	CDH23	1				
Q6ZTQ4	Cadherin-related family member 3	CDHR3	2				
A6H8M9	Cadherin-related family member 4	CDHR4	1				
Q99828	Calcium and integrin-binding protein 1	CIB1	1				X
Q14CN2	Calcium-activated chloride channel regulator 4	CLCA4	1				
Q9Y376	Calcium-binding protein 39	CAB39	3	X			
P62158	Calmodulin	CALM1	1				
P04632	Calpain small subunit 1	CAPNS1	1				X
O15484	Calpain-5	CAPN5	5	X			
P27797	Calreticulin	CALR	2			X	X
P17612	cAMP-dependent protein kinase catalytic subunit alpha	PRKAC	1				X
Q9GZX3	Carbohydrate sulfotransferase 6	CHST6	1				
P00918	Carbonic anhydrase 2	CA2	1				X
Q9GZU7	Carboxy-terminal domain RNA polymerase II polypeptide A small phosphatase 1	CTDSP1	1				
Q9UKL3	CASP8-associated protein 2	CASP8AP2	1				
P31944	Caspase-14	CASP14	2				
P04040	Catalase	CAT	2	X			X
P07858	Cathepsin B	CTSB	1	X		X	X
P07339	Cathepsin D	CTSD	1			X	X
P08311	Cathepsin G	CTSG	7	X		X	X

Accession #	Description	Gene name	# Peptides	Identified in both pools	Core exosome proteome	Membrane-bound vesicle proteins	Immune-related proteins
P11717	Cation-independent mannose-6-phosphate receptor	IGF2R	1			X	X
Q9NNX6	CD209 antigen	CD209	1				X
Q9Y5K6	CD2-associated protein	CD2AP	2	X			X
P29965	CD40 ligand	CD40LG	1				X
O43866	CD5 antigen-like	CD5L	3	X			X
P13987	CD59 glycoprotein	CD59	2	X	X		X
P08962	CD63 antigen	CD63	1		X	X	X
P21926	CD9 antigen	CD9	1	X	X	X	X
P60953	Cell division control protein 42 homolog	CDC42	1		X		X
P00450	Ceruloplasmin	CP	9	X			
Q7LBR1	Charged multivesicular body protein 1b	CHMP1B	1				
O43633	Charged multivesicular body protein 2a	CHMP2A	1				
Q9H444	Charged multivesicular body protein 4b	CHMP4B	1				
O00299	Chloride intracellular channel protein 1	CLIC1	3	X	X		X
O95833	Chloride intracellular channel protein 3	CLIC3	1				
Q9Y696	Chloride intracellular channel protein 4	CLIC4	1			X	X
Q96NY7	Chloride intracellular channel protein 6	CLIC6	5	X			
Q8IWA5	Choline transporter-like protein 2	SLC44A2	2	X			
Q53GD3	Choline transporter-like protein 4	SLC44A4	2	X			
Q00610	Clathrin heavy chain 1	CLTC	2		X	X	
P09497	Clathrin light chain B	CLTB	1	X		X	
P10909	Clusterin	CLU	12	X	X	X	X
P12259	Coagulation factor V	F5	3	X		X	X
P53618	Coatomer subunit beta	COPB1	1			X	
P23528	Cofilin-1	CFL1	3	X	X		
Q8NCX0	Coiled-coil domain-containing protein 150	CCDC150	1				
P25940	Collagen alpha-3(V) chain	COL5A3	1				
P02746	Complement C1q subcomponent subunit B	C1QB	1				
P06681	Complement C2	C2	2				X
P01024	Complement C3	C3	29	X			X

Accession #	Description	Gene name	# Peptides	Identified in both pools	Core exosome proteome	Membrane-bound vesicle proteins	Immune-related proteins
P0C0L4	Complement C4-A	C4A	5	X			X
P01031	Complement C5	C5	1				X
P13671	Complement component C6	C6	2	X			X
P07358	Complement component C8 beta chain	C8B	1	X			X
P00751	Complement factor B	CFB	3	X			X
P08603	Complement factor H	CFH	3	X			X
Q9BPX3	Condensin complex subunit 3	NCAPG	1				
P22528	Cornifin-B	SPRR1B	1				
Q9UBG3	Cornulin	CRNN	1	X			
P31146	Coronin-1A	CORO1A	1			X	X
Q9BUF7	Crumbs protein homolog 3	CRB3	1	X			
Q13616	Cullin-1	CUL1	1				
P04080	Cystatin-B	CSTB	3	X			
P01034	Cystatin-C	CST3	1				X
P01036	Cystatin-S	CST4	1				
P50238	Cysteine-rich protein 1	CRIP1	1				
P16562	Cysteine-rich secretory protein 2	CRISP2	1				
Q9UGM3	Deleted in malignant brain tumors 1 protein	DMBT1	24	X		X	X
P81605	Dermcidin	DCD	1				
Q6E0U4	Dermokine	DMKN	1				
Q08554	Desmocollin-1	DSC1	4	X			X
Q02413	Desmoglein-1	DSG1	4	X			
P32926	Desmoglein-3	DSG3	1	X			X
P15924	Desmoplakin	DSP	2				
P60981	Destrin	DSTN	1				
P27487	Dipeptidyl peptidase 4	DPP4	7	X	X	X	X
O14672	Disintegrin and metalloproteinase domain-containing protein 10	ADAM10	1			X	X
P49643	DNA primase large subunit	PRIM2	1				
Q13217	DnaJ homolog subfamily C member 3	DNAJC3	6	X			

Accession #	Description	Gene name	# Peptides	Identified in both pools	Core exosome proteome	Membrane-bound vesicle proteins	Immune-related proteins
Q9UJU6	Drebrin-like protein	DBNL	1	X			
Q9NRD8	Dual oxidase 2	DUOX2	3	X			
P11532	Dystrophin	DMD	1				X
O60291	E3 ubiquitin-protein ligase MGRN1	MGRN1	1	X			
O75355	Ectonucleoside triphosphate diphosphohydrolase 3	ENTPD3	1	X			
O14638	Ectonucleotide pyrophosphatase/phosphodiesterase family member 3	ENPP3	2	X			X
Q9H4M9	EH domain-containing protein 1	EHD1	3	X	X	X	
Q9H223	EH domain-containing protein 4	EHD4	1		X		
P68104	Elongation factor 1-alpha 1	EEF1A1	1		X		
P13639	Elongation factor 2	EEF2	3	X	X		
P14625	Endoplasmin	HSP90B1	2	X		X	X
P11678	Eosinophil peroxidase	EPX	2				X
Q12929	Epidermal growth factor receptor kinase substrate 8	EPS8	1	X			
Q8TE68	Epidermal growth factor receptor kinase substrate 8-like protein 1	EPS8L1	3	X			
Q9H6S3	Epidermal growth factor receptor kinase substrate 8-like protein 2	EPS8L2	1	X			
Q9GZV4	Eukaryotic translation initiation factor 5A-2	EIF5A2	1	X			
P56537	Eukaryotic translation initiation factor 6	EIF6	1				
Q96KP1	Exocyst complex component 2	EXOC2	1				
Q9GZZ8	Extracellular glycoprotein lacritin	LACRT	6	X		X	
P15311	Ezrin	EZR	27	X	X		
P47755	F-actin-capping protein subunit alpha-2	CAPZA2	1				
P47756	F-actin-capping protein subunit beta	CAPZB	2				
Q01469	Fatty acid-binding protein, epidermal	FABP5	2				X
P02671	Fibrinogen alpha chain	FGA	6	X		X	X
P02675	Fibrinogen beta chain	FGB	7	X		X	X
P02679	Fibrinogen gamma chain	FGG	9	X		X	X
Q14314	Fibroleukin	FGL2	1				X
P02751	Fibronectin	FN1	8	X	X	X	X

Accession #	Description	Gene name	# Peptides	Identified in both pools	Core exosome proteome	Membrane-bound vesicle proteins	Immune-related proteins
O00602	Ficolin-1	FCN1	1				
P20930	Filaggrin	FLG	15	X		X	
Q5D862	Filaggrin-2	FLG2	8	X			
P21333	Filamin-A	FLNA	6	X			X
O75369	Filamin-B	FLNB	1	X			
P30043	Flavin reductase	BLVRB	1				
P15328	Folate receptor alpha	FOLR1	1				
Q5H8C1	FRAS1-related extracellular matrix protein 1	FREM1	1				
P0C091	FRAS1-related extracellular matrix protein 3	FREM	5	X			
P09467	Fructose-1,6-bisphosphatase 1	FBP1	1				
P04075	Fructose-bisphosphate aldolase A	ALDOA	7	X	X	X	X
P17931	Galectin-3	LGALS3	5	X	X		X
Q08380	Galectin-3-binding protein	LGALS3BP	12	X	X		
P47929	Galectin-7	LGALS7	1				
P06396	Gelsolin	GSN	7	X	X		X
P11413	Glucose-6-phosphate 1-dehydrogenase	G6PD	1	X			
P06744	Glucose-6-phosphate isomerase	GPI	1				X
Q04609	Glutamate carboxypeptidase 2	FOLH1	4				
P15104	Glutamine synthetase	GLUL	2	X			
P08263	Glutathione S-transferase A1	GSTA1	5	X			
P09211	Glutathione S-transferase P	GSTP1	2		X		X
P04406	Glyceraldehyde-3-phosphate dehydrogenase	GAPDH	5	X	X		
P11217	Glycogen phosphorylase, muscle form	PYGM	7	X			
Q9NZH0	G-protein coupled receptor family C group 5 member B	GPRC5B	1			X	
Q9NQ84	G-protein coupled receptor family C group 5 member C	GPRC5C	1	X		X	
P01112	GTPase Hras	HRAS	1				X
P01116	GTPase Kras	KRAS	1	X			X
P62826	GTP-binding nuclear protein Ran	RAN	1	X	X	X	

Accession #	Description	Gene name	# Peptides	Identified in both pools	Core exosome proteome	Membrane-bound vesicle proteins	Immune-related proteins
P63096	Guanine nucleotide-binding protein G(i) subunit alpha-1	GNAI1	1				
P04899	Guanine nucleotide-binding protein G(i) subunit alpha-2	GNAI2	1		X		X
Q9UBI6	Guanine nucleotide-binding protein G(I)/G(S)/G(O) subunit gamma-12	GNG12	1				
P63218	Guanine nucleotide-binding protein G(I)/G(S)/G(O) subunit gamma-5	GNG5	1				
O60262	Guanine nucleotide-binding protein G(I)/G(S)/G(O) subunit gamma-7	GNG7	1				
P62879	Guanine nucleotide-binding protein G(I)/G(S)/G(T) subunit beta-2	GNB2	4		X		
P08754	Guanine nucleotide-binding protein G(k) subunit alpha	GNAI3	1	X	X	X	X
P50148	Guanine nucleotide-binding protein G(q) subunit alpha	GNAQ	4	X	X		X
P63092	Guanine nucleotide-binding protein G(s) subunit alpha isoforms short	GNAS	1		X		X
P29992	Guanine nucleotide-binding protein subunit alpha-11	GNA11	2		X		X
P00738	Haptoglobin	HP	16	X			X
P00739	Haptoglobin-related protein	HPR	8				
P08107	Heat shock 70 kDa protein 1A/1B	HSPA1A	6	X	X		
P34932	Heat shock 70 kDa protein 4	HSPA4	1				X
P11142	Heat shock cognate 71 kDa protein	HSPA8	8	X	X	X	X
P04792	Heat shock protein beta-1	HSPB1	1	X			
P07900	Heat shock protein HSP 90-alpha	HSP90AA1	3	X	X	X	X
P69905	Hemoglobin subunit alpha	HBA1	4	X			
P68871	Hemoglobin subunit beta	HBB	6				X
P02790	Hemopexin	HPX	2				X
P05546	Heparin cofactor 2	SERPIND1	1				X
P52790	Hexokinase-3	HK3	1				
Q13946	High affinity cAMP-specific 3',5'-cyclic phosphodiesterase 7A	PDE7A	1				X
P26583	High mobility group protein B2	HMGB2	1				
P42357	Histidine ammonia-lyase	HAL	3	X			
Q9NQE9	Histidine triad nucleotide-binding protein 3	HINT3	1				
P04196	Histidine-rich glycoprotein	HRG	2			X	X

Accession #	Description	Gene name	# Peptides	Identified in both pools	Core exosome proteome	Membrane-bound vesicle proteins	Immune-related proteins
P07305	Histone H1.0	H1F0	2				
P10412	Histone H1.4	HIST1H1E	4				
P16401	Histone H1.5	HIST1H1B	2				
Q96KK5	Histone H2A type 1-H	HIST1H2AH	4	X			
P0C0S5	Histone H2A.Z	H2AFZ	2				
O60814	Histone H2B type 1-K	HIST1H2BK	5	X			
Q6NXT2	Histone H3.3C	H3F3C	2				
P62805	Histone H4	HIST1H4A	8	X	X		
P30443	HLA class I histocompatibility antigen, A-1 alpha chain	HLA-A	1		X	X	X
P79483	HLA class II histocompatibility antigen, DR beta 3 chain	HLA-DRB3	2				X
Q86YZ3	Hornerin	HRNR	13	X			
Q9Y4L1	Hypoxia up-regulated protein 1	HYOU1	1				X
P01876	Ig alpha-1 chain C region	IGHA1	22	X			X
P01877	Ig alpha-2 chain C region	IGHA2	16	X			
P01880	Ig delta chain C region	IGHD	2				
P01857	Ig gamma-1 chain C region	IGHG1	5	X			X
P01860	Ig gamma-3 chain C region	IGHG3	6	X			X
P01861	Ig gamma-4 chain C region	IGHG4	2				
P01743	Ig heavy chain V-I region HG3		1	X			
P01825	Ig heavy chain V-II region NEWM		1	X			
P04438	Ig heavy chain V-II region SESS		2	X			
P01766	Ig heavy chain V-III region BRO		3				
P01782	Ig heavy chain V-III region DOB		1				
P01781	Ig heavy chain V-III region GAL		2				
P01765	Ig heavy chain V-III region TIL		4				
P01834	Ig kappa chain C region	IGKC	10	X			
P01597	Ig kappa chain V-I region DEE		4	X			

Accession #	Description	Gene name	# Peptides	Identified in both pools	Core exosome proteome	Membrane-bound vesicle proteins	Immune-related proteins
P01602	Ig kappa chain V-I region HK102 (Fragment)	IGKV1-5	2	X			
P01605	Ig kappa chain V-I region Lay		1				
P04431	Ig kappa chain V-I region Walker		1	X			X
P06310	Ig kappa chain V-II region RPMI 6410		2	X			
P01617	Ig kappa chain V-II region TEW		2				
P01621	Ig kappa chain V-III region NG9 (Fragment)		2				
P01623	Ig kappa chain V-III region WOL		3				
P06312	Ig kappa chain V-IV region (Fragment)	IGKV4	3				
P04211	Ig lambda chain V region 4A		1	X			
P01701	Ig lambda chain V-I region NEW		2				
P01702	Ig lambda chain V-I region NIG-64		3	X			
P04208	Ig lambda chain V-I region WAH		1	X			
P01710	Ig lambda chain V-II region BO		1				
P01706	Ig lambda chain V-II region BOH		1				
P01708	Ig lambda chain V-II region BUR		2	X			
P01709	Ig lambda chain V-II region MGC		2	X			
P01705	Ig lambda chain V-II region NEI		2	X			
P80748	Ig lambda chain V-III region LOI		2				
P01714	Ig lambda chain V-III region SH		1	X			
P01715	Ig lambda chain V-IV region Bau		1	X			
P0CG05	Ig lambda-2 chain C regions	IGLC2	9	X			
P0CG06	Ig lambda-3 chain C regions	IGLC3	8	X			
A0M8Q6	Ig lambda-7 chain C region	IGLC7	7	X			
P01871	Ig mu chain C region	IGHM	16	X			X
Q9Y6R7	IgGFc-binding protein	FCGBP	37	X			
P01591	Immunoglobulin J chain	IGJ	9	X			X
B9A064	Immunoglobulin lambda-like polypeptide 5	IGLL5	10	X			X
Q8N436	Inactive carboxypeptidase-like protein X2	CPXM2	1				

Accession #	Description	Gene name	# Peptides	Identified in both pools	Core exosome proteome	Membrane-bound vesicle proteins	Immune-related proteins
P12268	Inosine-5'-monophosphate dehydrogenase 2	IMPDH2	1				X
P19827	Inter-alpha-trypsin inhibitor heavy chain H1	ITIH1	1				
P19823	Inter-alpha-trypsin inhibitor heavy chain H2	ITIH2	3	X			
Q9UHD0	Interleukin-19	IL19	1				X
P07476	Involucrin	IVL	5	X			
O75874	Isocitrate dehydrogenase [NADP] cytoplasmic	IDH1	1	X			
O43240	Kallikrein-10	KLK10	1	X			
P13645	Keratin, type I cytoskeletal 10	KRT10	10	X	X		X
P02533	Keratin, type I cytoskeletal 14	KRT14	11				
P08779	Keratin, type I cytoskeletal 16	KRT16	9	X			
Q04695	Keratin, type I cytoskeletal 17	KRT17	8	X			X
P35527	Keratin, type I cytoskeletal 9	KRT9	12	X			
P04264	Keratin, type II cytoskeletal 1	KRT1	11	X			X
P35908	Keratin, type II cytoskeletal 2 epidermal	KRT2	7	X			
P13647	Keratin, type II cytoskeletal 5	KRT5	6	X	X		
P02538	Keratin, type II cytoskeletal 6A	KRT6	8	X			
O60938	Keratocan	KERA	1	X			X
Q5VWX1	KH domain-containing, RNA-binding, signal transduction-associated protein 2	KHDRBS2	1				
O60282	Kinesin heavy chain isoform 5C	KIF5C	1	X			
P01042	Kininogen-1	KNG1	1			X	X
P22079	Lactoperoxidase	LPO	3	X			
P02788	Lactotransferrin	LTF	61	X		X	X
Q04760	Lactoylglutathione lyase	GLO1	1				
P11047	Laminin subunit gamma-1	LAMC1	1				
Q9BXB1	Leucine-rich repeat-containing G-protein coupled receptor 4	LGR4	1				
P30740	Leukocyte elastase inhibitor	SERPINB1	3	X			X
Q14847	LIM and SH3 domain protein 1	LASP1	5	X			
P31025	Lipocalin-1	LCN1	2				
Q6UWW0	Lipocalin-15	LCN15	3	X			

Accession #	Description	Gene name	# Peptides	Identified in both pools	Core exosome proteome	Membrane-bound vesicle proteins	Immune-related proteins
Q9NZR2	Low-density lipoprotein receptor-related protein 1B	LRP1B	1	X			
Q6UXB3	Ly6/PLAUR domain-containing protein 2	LYPD2	1				
Q14210	Lymphocyte antigen 6D	LY6D	2				
P33241	Lymphocyte-specific protein 1	LSP1	1				X
Q14108	Lysosome membrane protein 2	SCARB2	1				
P11279	Lysosome-associated membrane glycoprotein 1	LAMP1	1	X	X	X	X
P61626	Lysozyme C	LYZ	8	X			X
Q96JB8	MAGUK p55 subfamily member 4	MPP4	1				
Q14764	Major vault protein	MVP	1		X		X
P40926	Malate dehydrogenase, mitochondrial	MDH2	1				
O75556	Mammaglobin-B	SCGB2A1	3	X			
A6NHS7	MANSC domain-containing protein ENSP00000370673		1				
Q96A59	MARVEL domain-containing protein 3	MARVELD3	1				
O95460	Matrilin-4	MATN4	2	X			
P14780	Matrix metalloproteinase-9	MMP9	1	X			X
P08582	Melanotransferrin	MFI2	2	X			
Q13421	Mesothelin	MSLN	2	X			X
Q15691	Microtubule-associated protein RP/EB family member 1	MAPRE1	1				
P21741	Midkine	MDK	2				X
P45984	Mitogen-activated protein kinase 9	MAPK9	1				X
P26038	Moesin	MSN	18	X	X	X	X
P08571	Monocyte differentiation antigen CD14	CD14	3	X			X
P15941	Mucin-1	MUC1	3	X	X		X
Q9H3R2	Mucin-13	MUC13	1				
Q8WXI7	Mucin-16	MUC16	10	X			
Q02817	Mucin-2	MUC2	1				
Q99102	Mucin-4	MUC4	3				
P98088	Mucin-5AC (Fragments)	MUC5AC	6				X

Accession #	Description	Gene name	# Peptides	Identified in both pools	Core exosome proteome	Membrane-bound vesicle proteins	Immune-related proteins
Q9HC84	Mucin-5B	MUC5B	19	X			
Q8TAX7	Mucin-7	MUC7	2				
P24158	Myeloblastin	PRTN3	1				X
Q96S97	Myeloid-associated differentiation marker	MYADM	1				
P05164	Myeloperoxidase	MPO	8	X		X	X
Q9NZM1	Myoferlin	MYOF	1			X	
P60660	Myosin light polypeptide 6	MYL6	2				
P35579	Myosin-9	MYH9	37	X	X		X
O43795	Myosin-Ib	MYO1B	1				
O00159	Myosin-Ic	MYO1C	3				
O94832	Myosin-Id	MYO1D	3	X			
Q9NQX4	Myosin-Vc	MYO5C	1				
Q92614	Myosin-XVIIIa	MYO18A	1				
P29966	Myristoylated alanine-rich C-kinase substrate	MARCKS	1				
O14745	Na(+)/H(+) exchange regulatory cofactor NHE-RF1	SLC9A3R1	9	X	X		
Q15599	Na(+)/H(+) exchange regulatory cofactor NHE-RF2	SLC9A3R2	5	X			
Q56NI9	N-acetyltransferase ESCO2	ESCO2	1	X			
Q8N205	Nesprin-4	C19orf46	1				
O95185	Netrin receptor UNC5C	UNC5C	1				
Q09666	Neuroblast differentiation-associated protein AHNAK	AHNAK	1				X
Q14697	Neutral alpha-glucosidase AB	GANAB	7	X		X	
Q15758	Neutral amino acid transporter B(0)	SLC1A5	1			X	
P59665	Neutrophil defensin 1	DEFA1	2	X		X	
P08246	Neutrophil elastase	ELANE	1	X		X	X
P80188	Neutrophil gelatinase-associated lipocalin	LCN2	1				X
Q96TA1	Niban-like protein 1	FAM129B	2				
P35228	Nitric oxide synthase, inducible	NOS2	10	X			X
Q8IVI9	Nostrin	NOSTRIN	1			X	
Q9GZM8	Nuclear distribution protein nudE-like 1	NDEL1	1				

Accession #	Description	Gene name	# Peptides	Identified in both pools	Core exosome proteome	Membrane-bound vesicle proteins	Immune-related proteins
Q02818	Nucleobindin-1	NUCB1	4	X			
P80303	Nucleobindin-2	NUCB2	4	X			
Q9H173	Nucleotide exchange factor SIL1	SIL1	1				
P55259	Pancreatic secretory granule membrane major glycoprotein GP2	GP2 PE	2				
O14908	PDZ domain-containing protein GIPC1	GIPC1	1	X		X	
Q13113	PDZK1-interacting protein 1	PDZK1IP1	2	X			
P19021	Peptidyl-glycine alpha-amidating monooxygenase	PAM	1			X	
P62937	Peptidyl-prolyl cis-trans isomerase A	PPIA	2	X	X		X
P23284	Peptidyl-prolyl cis-trans isomerase B	PPIB	1	X		X	X
O43447	Peptidyl-prolyl cis-trans isomerase H	PPIH	1				
Q06830	Peroxiredoxin-1	PRDX1	3	X	X	X	X
P32119	Peroxiredoxin-2	PRDX2	1		X		X
Q13162	Peroxiredoxin-4	PRDX4	2	X			
Q5SXH7	PH domain-containing protein C10orf81	C10orf81	1				
Q96S96	Phosphatidylethanolamine-binding protein 4	PEBP4	1				
Q8TBX8	Phosphatidylinositol-5-phosphate 4-kinase type-2 gamma	PIP4K2C	1				
P00558	Phosphoglycerate kinase 1	PGK1	4		X		
P18669	Phosphoglycerate mutase 1	PGAM1	5	X			
P14555	Phospholipase A2, membrane associated	PLA2G2A	2	X		X	X
O15162	Phospholipid scramblase 1	PLSCR1	1				X
P55058	Phospholipid transfer protein	PLTP	2	X			X
P05155	Plasma protease C1 inhibitor	SERPING1	1			X	X
P00747	Plasminogen	PLG	4	X		X	X
P07359	Platelet glycoprotein Ib alpha chain	GP1BA	1				X
P43034	Platelet-activating factor acetylhydrolase IB subunit alpha	PAFAH1B1	1				X
Q494U1	Pleckstrin homology domain-containing family N member 1	PLEKHN1	1				
O00592	Podocalyxin	PODXL	1				X

Accession #	Description	Gene name	# Peptides	Identified in both pools	Core exosome proteome	Membrane-bound vesicle proteins	Immune-related proteins
Q460N3	Poly [ADP-ribose] polymerase 15	PARP15	1				
P01833	Polymeric immunoglobulin receptor	PIGR	31	X	X	X	X
Q8IXK2	Polypeptide N-acetylgalactosaminyltransferase 12	GALNT12	1				
P0CG47	Polyubiquitin-B	UBB	3	X	X	X	
Q9P1Z3	Potassium/sodium hyperpolarization-activated cyclic nucleotide-gated channel 3	HCN3	1				
Q6S8J3	POTE ankyrin domain family member E	POTE	7				
P02545	Prelamin-A/C	LMNA	1	X			
Q13206	Probable ATP-dependent RNA helicase DDX10	DDX10	1				
Q8WUW1	Probable protein BRICK1	C3orf10	1				
Q02809	Procollagen-lysine,2-oxoglutarate 5-dioxygenase 1	PLOD1	2	X			
P07737	Profilin-1	PFN1	4	X	X		X
Q8WUM4	Programmed cell death 6-interacting protein	PDCD6IP	13	X	X	X	
P12273	Prolactin-inducible protein	PIP	2	X			
Q99935	Proline-rich protein 1	PROL1	3	X			
Q16378	Proline-rich protein 4	PRR4	3	X			
O43490	Prominin-1	PROM1	9	X			
Q8N271	Prominin-2	PROM2	1			X	
Q16651	Prostasin	PRSS8	1				
Q9UL46	Proteasome activator complex subunit 2	PSME2	1	X			X
P25786	Proteasome subunit alpha type-1	PSMA1	1				
P25787	Proteasome subunit alpha type-2	PSMA2	2	X			
P25788	Proteasome subunit alpha type-3	PSMA3	1				
P60900	Proteasome subunit alpha type-6	PSMA6	1				
P28070	Proteasome subunit beta type-4	PSMB4	1				
P28074	Proteasome subunit beta type-5	PSMB5	2				X
P28072	Proteasome subunit beta type-6	PSMB6	1	X			
P28062	Proteasome subunit beta type-8	PSMB8	1	X			X
Q8TCG1	Protein CIP2A	KIAA1524	1				
O00622	Protein CYR61	CYR61	1				X

Accession #	Description	Gene name	# Peptides	Identified in both pools	Core exosome proteome	Membrane-bound vesicle proteins	Immune-related proteins
Q9P219	Protein Daple	CCDC88C	1				
P07237	Protein disulfide-isomerase	P4HB	5	X	X	X	X
Q96MK3	Protein FAM20A	FAM20A	1				
Q9UNF0	Protein kinase C and casein kinase substrate in neurons protein 2	PACSIN2	1			X	
Q9UN36	Protein NDRG2	NDRG2	1				
Q9UD71	Protein phosphatase 1 regulatory subunit 1B	PPP1R1B	1	X			
Q16821	Protein phosphatase 1 regulatory subunit 3A	PPP1R3A	1				
Q5SGD2	Protein phosphatase 1L	PPM1L	1				
P31949	Protein S100-A11	S100A11	1	X			
P80511	Protein S100-A12	S100A12	1				X
Q9HCY8	Protein S100-A14	S100A14	1	X			X
Q96FQ6	Protein S100-A16	S100A16	3	X			
P29034	Protein S100-A2	S100A2	1				
P26447	Protein S100-A4	S100A4	2	X			X
P05109	Protein S100-A8	S100A8	10	X			X
P06702	Protein S100-A9	S100A9	9	X			X
P22061	Protein-L-isoaspartate(D-aspartate) O-methyltransferase	PCMT1	1				
P00734	Prothrombin	F2	1	X			X
Q9NYQ8	Protocadherin Fat 2	FAT2	1				
A6NMY6	Putative annexin A2-like protein	ANXA2P2	3			X	
P0C874	Putative FAM75-like protein FLJ44082		1				
B2RPK0	Putative high mobility group protein B1-like 1	HMGB1L1	1	X			
Q2M2H8	Putative maltase-glucoamylase-like protein LOC93432		2				
B8ZZ34	Putative protein shisa-8	SHISA8	1	X			
A6NJ16	Putative V-set and immunoglobulin domain-containing protein 6	VSIG6	1				
P14618	Pyruvate kinase isozymes M1/M2	PKM2	4	X	X		
P50395	Rab GDP dissociation inhibitor beta	GDI2	1		X		

Accession #	Description	Gene name	# Peptides	Identified in both pools	Core exosome proteome	Membrane-bound vesicle proteins	Immune-related proteins
P35241	Radixin	RDX	17	X	X		
O95398	Rap guanine nucleotide exchange factor 3	RAPGEF3	1				X
P46940	Ras GTPase-activating-like protein IQGAP1	IQGAP1	6	X	X		
P63000	Ras-related C3 botulinum toxin substrate 1	RAC1	2	X	X	X	X
P15153	Ras-related C3 botulinum toxin substrate 2	RAC2	2				X
P61026	Ras-related protein Rab-10	RAB10	1		X		
P62491	Ras-related protein Rab-11A	RAB11A	1	X	X	X	
P51153	Ras-related protein Rab-13	RAB13	1		X	X	
Q9ULC3	Ras-related protein Rab-23	RAB23	1				
P57735	Ras-related protein Rab-25	RAB25	1			X	
P20339	Ras-related protein Rab-5A	RAB5A	1	X	X	X	X
P61006	Ras-related protein Rab-8A	RAB8A	1				
P11234	Ras-related protein Ral-B	RALB	1	X			X
A6NIZ1	Ras-related protein Rap-1b-like protein		3				
Q12913	Receptor-type tyrosine-protein phosphatase eta	PTPRJ	1				X
O75787	Renin receptor	ATP6AP2	1	X			
Q6XPR3	Repetin	RPTN	2				
P00352	Retinal dehydrogenase 1	ALDH1A1	4				
P49788	Retinoic acid receptor responder protein 1	RARRES1	1	X			
Q8NFJ5	Retinoic acid-induced protein 3	GPRC5A	2	X		X	
P52565	Rho GDP-dissociation inhibitor 1	ARHGDIA	2	X			X
P52566	Rho GDP-dissociation inhibitor 2	ARHGDIB	2			X	X
Q07960	Rho GTPase-activating protein 1	ARHGAP1	1	X			X
Q96QB1	Rho GTPase-activating protein 7	DLC1	1				
Q13464	Rho-associated protein kinase 1	ROCK1	2				X
P34096	Ribonuclease 4	RNASE4	1				
O00584	Ribonuclease T2	RNASET2	1				
Q5GAN4	Ribonuclease-like protein 12	RNASE12	1				
Q9P2E9	Ribosome-binding protein 1	RRBP1	5	X			
Q86SE5	RNA-binding Raly-like protein	RALYL	1				

Accession #	Description	Gene name	# Peptides	Identified in both pools	Core exosome proteome	Membrane-bound vesicle proteins	Immune-related proteins
Q8N474	Secreted frizzled-related protein 1	SFRP1	5	X			X
O95968	Secretoglobin family 1D member 1	SCGB1D1	3	X			
Q02383	Semenogelin-2	SEMG2	10	X		X	
Q92743	Serine protease HTRA1	HTRA1	5	X			
Q9Y6E0	Serine/threonine-protein kinase 24	STK24	1				
Q9Y5S2	Serine/threonine-protein kinase MRCK beta	CDC42BPB	1				
Q7L7X3	Serine/threonine-protein kinase TAO1	TAOK1	2	X			
Q9H2K8	Serine/threonine-protein kinase TAO3	TAOK3	2				
P36873	Serine/threonine-protein phosphatase PP1-gamma catalytic subunit	PPP1CC	1	X			
P02787	Serotransferrin	TF	24	X		X	X
P29508	Serpin B3	SERPINB3	8	X			X
P50452	Serpin B8	SERPINB8	1				
P02768	Serum albumin	ALB	24	X	X	X	X
P02735	Serum amyloid A protein	SAA1	2	X			X
Q9H788	SH2 domain-containing protein 4A	SH2D4A	1				
Q9UBC9	Small proline-rich protein 3	SPRR3	1				
Q9UN76	Sodium- and chloride-dependent neutral and basic amino acid transporter B(0+)	SLC6A14	4	X			
P13866	Sodium/glucose cotransporter 1	SLC5A1	1				
P48764	Sodium/hydrogen exchanger 3	SLC9A3	1				
Q8N695	Sodium-coupled monocarboxylate transporter 1	SLC5A8	2	X			
O95436	Sodium-dependent phosphate transport protein 2B	SLC34A	1				
Q16348	Solute carrier family 15 member 2	SLC15A2	4	X			
Q9HAS3	Solute carrier family 28 member 3	SLC28A3	2	X			
Q00796	Sorbitol dehydrogenase	SORD	3	X			
P30626	Sorcin	SRI	1				
Q96RF0	Sorting nexin-18	SNX18	2			X	
Q14515	SPARC-like protein 1	SPARCL1	1				
Q9BW04	Specifically androgen-regulated gene protein	SARG	1	X			
Q01082	Spectrin beta chain, brain 1	SPTBN1	1				

Accession #	Description	Gene name	# Peptides	Identified in both pools	Core exosome proteome	Membrane-bound vesicle proteins	Immune-related proteins
Q9H254	Spectrin beta chain, brain 3	SPTBN4	1	X			
Q14247	Src substrate cortactin	CTTN	3	X			X
Q9NQZ5	StAR-related lipid transfer protein 7, mitochondrial	STARD	1				
Q9H2G2	STE20-like serine/threonine-protein kinase	SLK	2	X			
P09238	Stromelysin-2	MMP10	2				X
O00391	Sulfhydryl oxidase 1	QSOX1	3	X			
P04179	Superoxide dismutase [Mn], mitochondrial	SOD2	1				X
Q6UWP8	Suprabasin	SBSN	2	X			
Q99536	Synaptic vesicle membrane protein VAT-1 homolog	VAT1	1				
O00161	Synaptosomal-associated protein 23	SNAP23	1			X	
Q13277	Syntaxin-3	STX3	2	X		X	
Q15833	Syntaxin-binding protein 2	STXBP2	3	X		X	
O00560	Syntenin-1	SDCBP	2		X	X	
Q9H190	Syntenin-2	SDCBP2	1	X			
Q9Y490	Talin-1	TLN1	1				X
Q6IQ55	Tau-tubulin kinase 2	TTBK2	1				
P17987	T-complex protein 1 subunit alpha	TCP1	1				
P48643	T-complex protein 1 subunit epsilon	CCT5	1				
P40227	T-complex protein 1 subunit zeta	CCT6A	1				
P24821	Tenascin	TNC	2				X
Q96AE7	Tetratricopeptide repeat protein 17	TTC17	1				
P10599	Thioredoxin	TXN	3	X			X
P07996	Thrombospondin-1	THBS1	3	X	X	X	X
P19971	Thymidine phosphorylase	TYMP	1				X
O75674	TOM1-like protein 1	TOM1L1	1				
P20061	Transcobalamin-1	TCN1	1				
P35711	Transcription factor SOX-5	SOX5	1	X			
Q15582	Transforming growth factor-beta-induced protein ig-h3	TGFBI	1				X
P61586	Transforming protein RhoA	RHOA	2	X			X

Accession #	Description	Gene name	# Peptides	Identified in both pools	Core exosome proteome	Membrane-bound vesicle proteins	Immune-related proteins
P29401	Transketolase	TKT	5	X			X
O15321	Transmembrane 9 superfamily member 1	TM9SF1	1				
Q7Z404	Transmembrane channel-like protein 4	TMC4	2	X			
Q6UXY8	Transmembrane channel-like protein 5	TMC5	8	X			
Q9BUB7	Transmembrane protein 70, mitochondrial	TMEM70	1				
P02766	Transthyretin	TTR	1				
Q07654	Trefoil factor 3	TFF3	2	X		X	X
P60174	Triosephosphate isomerase	TPI1	1	X	X		X
P68366	Tubulin alpha-4A chain	TUBA4A	2				
P07437	Tubulin beta chain	TUBB	2				X
P50591	Tumor necrosis factor ligand superfamily member 10	TNFSF10	2				X
Q99816	Tumor susceptibility gene 101 protein	TSG101	1		X		
P09758	Tumor-associated calcium signal transducer 2	TACSTD2	1				
O15327	Type II inositol-3,4-bisphosphate 4-phosphatase	INPP4B	1				
P07948	Tyrosine-protein kinase Lyn	LYN	1				X
Q8WUN7	Ubiquitin domain-containing protein 2	UBTD2	1				
P22314	Ubiquitin-like modifier-activating enzyme 1	UBA1	2	X			
Q8NFL0	UDP-GlcNAc:betaGal beta-1,3-N-acetylglucosaminyltransferase 7	B3GNT7	1				
Q96A22	Uncharacterized protein C11orf52	C11orf52	1				
Q9H246	Uncharacterized protein C1orf21	C1orf21	1	X			
Q6NV74	Uncharacterized protein C2orf55	C2orf55	1				
Q86YA3	Uncharacterized protein C4orf21	C4orf21	1				
Q5T0Z8	Uncharacterized protein C6orf132	C6orf132	3				
Q8N8K9	Uncharacterized protein KIAA1958	KIAA1958	1				
Q6P5S2	UPF0762 protein C6orf58	C6orf58	5	X			
P11684	Uteroglobin	SCGB1A1	1	X			X
Q709C8	Vacuolar protein sorting-associated protein 13C	VPS13C	1				
Q9UK41	Vacuolar protein sorting-associated protein 28 homolog	VPS28	1		X		

Accession #	Description	Gene name	# Peptides	Identified in both pools	Core exosome proteome	Membrane-bound vesicle proteins	Immune-related proteins
O75083	WD repeat-containing protein 1	WDR1	3	X	X		
Q6UXB2	VEGF co-regulated chemokine 1	CXCL17	4	X			X
Q9UIW0	Ventral anterior homeobox 2	VAX2	1				
P49748	Very long-chain specific acyl-CoA dehydrogenase, mitochondrial	ACADVL	1				
Q9BV40	Vesicle-associated membrane protein 8	VAMP8	2	X		X	X
P02774	Vitamin D-binding protein	GC	3	X			X
P07225	Vitamin K-dependent protein S	PROS1	2	X		X	X
P04004	Vitronectin	VTN	1				X
Q6PCB0	von Willebrand factor A domain-containing protein 1	VWA1	2	X			
Q9BRH9	Zinc finger protein 251	ZNF251	1				
O75373	Zinc finger protein 737	ZNF737	1				
P25311	Zinc-alpha-2-glycoprotein	AZGP1	3	X			
Q96DA0	Zymogen granule protein 16 homolog B	ZG16B	4	X			
